# An option space approach to wood use: Providing structural timber for buildings while safeguarding forest integrity

**DOI:** 10.1016/j.isci.2025.113472

**Published:** 2025-09-02

**Authors:** Simone Gingrich, Sarah Matej, Karl-Heinz Erb, Helmut Haberl, Julia Le Noë, Lisa Kaufmann, Andreas Magerl, Anke Schaffartzik, Dominik Wiedenhofer, Stefan Pauliuk

**Affiliations:** 1 Institute of Social Ecology, Department of Economics and Social Sciences, BOKU University, 1070 Vienna, Austria; 2 Department of Environmental Science and Policy, Central European University, 1100 Vienna, Austria; 3 Institut de Recherche pour le Développement, 75252 Paris, France; 4 Industrial Ecology Group, University of Freiburg, 79106 Freiburg, Germany

**Keywords:** Earth sciences, Environmental science, Global change, Environmental management, Environmental policy, Nature conservation, Natural resources, Forestry

## Abstract

Wood use is crucial for climate-change mitigation, but strategies range from increasing harvest to conserving forests. To reconcile contradictions, we conceptualize an option space that considers both social and ecological thresholds. We couple the material flow model RECC and the forest model CRAFT to quantify the option space for wood use in the global building sector and current forest areas from 2020 to 2050. We juxtapose four demand scenarios with four supply scenarios that meet material and ecosystem service thresholds, respectively. In 12 of the 16 resulting scenario combinations, supply exceeds demand. They differ in regional self-sufficiency (6–9 out of ten world regions), average primary wood availability beyond structural timber use (0.2–1.4 GtCyr^−1^), and overall climate impacts (2.0–8.0 GtCO_2_eqyr^−1^). Substantially increasing wood intensity in buildings within ecological limits is only feasible in a low floorspace scenario with increasing circularity, emphasizing the need for nuance in claims regarding the sustainability of wood use.

## Highlights

• We conceptualize sustainable wood use in an option space approach
• We independently model global sustainable wood supply and demand in buildings by 2050
• Increasing timber in construction is feasible only in a low floorspace scenario
• Reduced floorspace mitigates climate impacts and leaves more wood for other uses

## Graphical abstract

**Figure:**
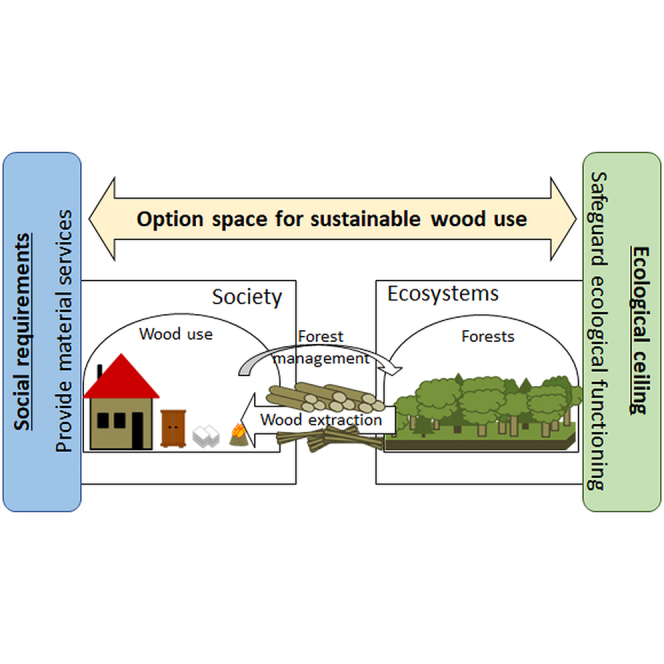

## Introduction

Wood plays an integral role in multiple climate-change mitigation strategies. There is a general consensus that ending deforestation and forest degradation, i.e., conserving woody biomass in forests, is imperative for mitigating the climate crisis^1^^,^^2^^,^^3^: in most climate strategies, forests are expected to remain carbon (C) sinks.^4^^,^^5^ Beyond that, political and academic debates continue over the question to which extent increased or reduced use of wood as material or energy carrier can contribute to climate-change mitigation. Disagreements prevail both in conceptual and empirical terms about whether, under which conditions and to which extent the emission savings expected from replacing emission-intensive products with wood use outweigh the impacts of wood extraction on the carbon balance of forests.^6^^,^^7^ Arguments are particularly heated when it comes to the climate impacts of forest bioenergy,^8^^,^^9^^,^^10^ because about half of all globally removed wood is currently used as woodfuel.^11^ Debates over the potential trade-offs associated to harvesting wood for material uses have intensified more recently, juxtaposing the climate benefits of material use of timber with the negative ecological effects of increasing timber harvest.^12^^,^^13^^,^^14^ Integrative approaches that enable assessing both, the positive and negative climate effects of wood use, are therefore urgently needed.

On the one hand, material uses of wood have the potential double benefit of substituting energy- and emissions-intensive products, while also accumulating carbon in the built environment.^15^^,^^16^ This is particularly true in construction, where high substitution effects (e.g., of steel-reinforced concrete) coincide with long storage times.^17^^,^^18^ Therefore, a number of studies propose construction wood as a substantial and viable climate solution (for a meta-study, see Himes and Busby^19^). Estimates of the contribution of construction timber to sustainable resource provision vary across authors: Johnston and Radeloff^20^ quantify future C accumulation in harvested wood products to peak around 2030 at a rate of 0.3–0.4 GtCO_2eq_yr^−1^, depending on the socio-economic scenario, and argue that by accumulating the equivalent of <1% of global greenhouse gas emissions, the global climate-change mitigation contribution of harvested wood products is only minor. By contrast, Mishra et al.^21^ highlight the potential of construction timber to provide housing through the establishment of global “timber cities”. They estimate that by 2100, new timber buildings could house 90% of additional urban residents globally, storing in total 106 GtCO_2eq,_ while high levels of wood provision could be achieved by expanding tree plantations into non-agricultural land. Despite these variations in assessments, using timber in construction is generally regarded as a climate-friendly option.^22^

On the other hand, there is increasing evidence that large C sequestration potentials could be realized in forests by reducing wood harvest in existing forests^23^ or allowing forest recovery on non-forest land,^24^ highlighting the ecological limits of intensifying timber extraction for material use. Large variations exist between estimates of maximum ecological forest C pool potentials, resulting from both conceptual differences and empirical uncertainties: At the higher end, Bastin et al.^25^ claim that ecosystems could hypothetically sequester up to 750 GtCO_2_ globally if the ecological tree restoration potential were to be reached through both expansion and thickening of tree cover. Erb et al.^26^ estimate that on existing forest land, the difference between currently prevailing (actual) biomass and its ecological maximum potential amounts to 488 GtCO_2_, with Mo et al.^27^ and Walker et al.^28^ finding similar values. At the lower end of the spectrum, Roebroek et al.^29^ estimate the potential of taking existing forests out of use at only 161 GtCO_2_. This wide range of current estimates of ecological C pool potentials in forest ecosystems is mainly due to the uncertain impact of natural disturbances such as droughts and forest fires. Further constraints in realizing potential ecological C stocks apply, among others, to their social and economic feasibility.^30^ Despite prevailing debates over its potential, safeguarding woody biomass accumulation in ecosystems plays a crucial role for reaching climate targets.^6^

An obvious trade-off exists between increasing timber harvest and sequestering C in ecosystems,^31^ even if global forest area might be expanded and annual woody increment increased to some extent through better management.^32^ National and regional case studies both in the Global North^13^^,^^33^^,^^34^ and the Global South^35^ show that reducing harvest impacts is an effective measure to increase the C accumulation in forests in the short to medium term, even when factoring in the climate-change mitigation effect of harvested wood products substituting for emission-intensive materials and products. In its section “Improved and Enhanced Use of Wood Products”, the recent IPCC report cautions that enhanced use of wood products may result in climate benefits or risks, depending on both the way the wood is used (e.g., its lifetime as material), and forest management.^3^

Surprisingly, however, the trade-off between wood provision and C sequestration in forest biomass remains entirely unaddressed in global assessments of land-based climate-change mitigation options such as those underlying IPCC scenarios.^2^^,^^36^ In part, this is due to the insufficient representation of the effects of wood harvest on forest C pools in many integrated assessment models.^37^ Recent analyses based on global forest sector models show that more effective forest management may contribute to growing forest C stocks, while simultaneously allowing for increasing harvest on the same land.^38^ By using market-oriented assumptions^20^^,^^38^^,^^39^ to model future dynamics of wood harvest and forest change under specific socio-economic scenarios (e.g., varying carbon prices), these studies do not address the provision of material services. Only recently, studies have started to address the challenge of meeting societal demand for wood products while remaining within ecological limits.^40^^,^^41^ In addition, a number of studies have conceptually and empirically advanced the understanding of climate impacts associated to wood harvest.^42^^,^^43^^,^^44^ Nevertheless, knowledge gaps prevail on delineating corridors of wood provision that meet material services while respecting ecological limits, and the overall climate impacts they entail.

**Figure 1:**
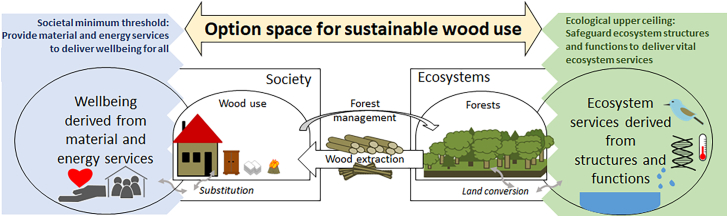
Conceptualization of the proposed option space for sustainable wood use

Here, we introduce the *option space for sustainable wood use*, defined as those combinations of wood demand and supply scenarios that ensure decent material service provision for all^45^^,^^46^ while safeguarding forest ecosystem integrity in terms of ecosystem functioning and services^47^^,^^48^ (Figure 1). Our framework builds on the “doughnut” metaphor for sustainable resource use,^49^ characterizing a safe and just operating space in which wellbeing for all is achieved without transgressing planetary boundaries. Previous work using the doughnut approach^50^^,^^51^ has approximated the multiple social and ecological sustainability challenges of resource use in general. We apply this approach to explore sustainable corridors for using one resource, i.e., wood, and one major material service, i.e., shelter provision in the building sector with structural timber. In line with previous research on biophysically feasible land-use options that meet both social and ecological sustainability criteria,^52^^,^^53^^,^^54^ we use the term “option space”. In our analysis, we use carbon stocks and flows in ecosystems and society as the key performance indicators and proxies for the main sustainability dimensions, material use in housing services, ecosystem integrity, and human impact on the climate.

By coupling a building sector model to a forest growth model, we assess the global option space for sustainable wood use in the global building sector (Figure S1 in supplemental methods). We directly compare the demand for industrial roundwood required for structural timber used in global buildings with the wood harvest from current (2020) forest areas during the period 2020–2050. Four scenarios are defined independently for wood supply and demand, leading to 16 different scenario combinations. The option space comprises those scenario combinations in which supply exceeds demand. Wood demand in the building sector, both structural timber and industrial roundwood, is given by four published scenarios^55^ generated with the RECC (resource efficiency–climate change) model.^56^ The four scenarios are defined by two levels of floorspace provision beyond a minimum threshold (i.e., business-as-usual (SSP2)^57^ and low energy and material demand (LEMD),^58^ denoted *Floorspace*^*+*^ and *Floorspace*^*-*^, respectively), as well as two different building material choices (increased or constant fraction of wood use in buildings (_*timber^+^* and *_timber*^*∼*^)). The two *Floorspace*^*-*^ scenarios further assume higher circularity in timber use (therefore, their acronyms additionally feature *_cascade^+^*). For details on the RECC model and its parameters, see STAR Methods section, key resources table, and supplemental methods. To quantify wood supply from current forest areas under ecological constraints, we use the parsimonious forest growth model CRAFT (CaRbon Accumulation in ForesT).^59^^,^^60^ We develop four scenarios of wood provision in CRAFT that safeguard ecosystem services by modulating wood harvest levels and forest growth rates. We implement an ecological integrity target (*EcoInt*), limiting harvest to 75% of gross annual increment (GAI),^61^ and a forest carbon sink target (*Csink*), limiting harvest to safeguard the technical potential of C accumulation in forest biomass.^36^ Achieving each of these targets is computed under two assumptions on future forest growth that build on forest dynamics reported by FAOstat in 1990–2020. We thus develop, for both ecological targets to be reached, a variant under continuously improving^32^^,^^62^ or non-improving^63^^,^^64^ forest growth conditions (_*growth^+^* and *_growth*^*∼*^, respectively). For details on the CRAFT model, see STAR Methods section, key resources table and supplemental methods. We first present the demand and supply scenarios separately and then, by juxtaposing the RECC and CRAFT scenarios, we delineate the option space for sustainable wood use. For each feasible scenario combination, we quantify the primary wood supply beyond uses in the building sector as the difference between sustainable wood supply and industrial roundwood demand for buildings. In addition, we assess the overall climate impact of each scenario combination, i.e., the fossil-fuel based greenhouse gas emissions of the building sector minus the net C accumulation in socio-economic timber pools and forest biomass. The aim of our analysis is to offer a conceptual approach and first empirical approximation for assessing the benchmarks for socially and ecologically sustainable wood provision and its climate impacts.

## Results

### Wood demand for housing services

**Figure 2:**
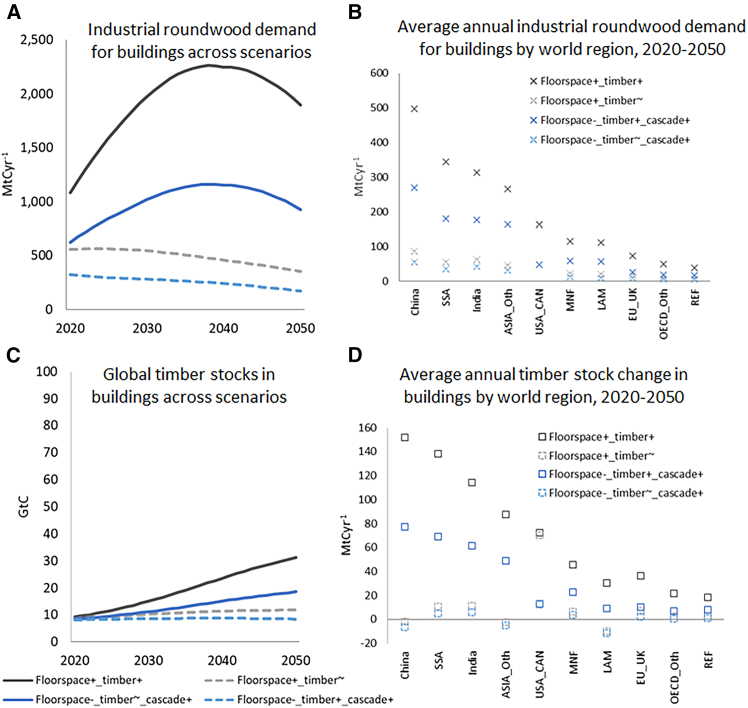
Projections of wood demand to meet material services in the building sector in the four scenarios increased floorspace provision (floorspace^+_^timber^−^), increased floorspace provision with increased timber in construction (floorspace^+^_timber^+^), reduced floorspace provision with increasingly cascadic timber use (floorspace^−^_timber^−^_cascade^+^), reduced floorspace provision with increasingly cascadic timber use and increased timber in construction (floorspace^−^_timber^+^_cascade^+^) (A) annual global industrial roundwood demand in the building sector across scenarios; (B) average annual industrial roundwood demand for buildings by world region, 2020–2050. For a list of the world regions aggregated, see Table S1 and data supplement.; (C) global timber C stocks buildings across scenarios (“in-use stocks”), (D) average annual timber C stock change in buildings by world region across scenarios in 2020–2050. For a list of the world regions aggregated, see Table S1 and data supplement.

Industrial roundwood for structural timber demand by global housing services in 2050, according to the results of the RECC model, differs by a factor >10, i.e., from 170 MtCyr^−1^ (*Floorspace*^*-*^_*_*_*timber*^*-*^_*_*_*cascade*^*+*^) to 1,900 MtCyr^−1^ (*Floorspace*^*+*^_*_*_*timber*^*+*^, Figure 2A). Scenarios with a constant wood share in construction (*Floorspace*^*-*^*_timber*^*∼*^*_cascade*^*+*^ and *Floorspace*^*+*^*_timber*^*∼*^) show an overall declining wood demand (53% and 64% of the 2020 value, respectively). Due to the lower inflow of industrial roundwood, these scenarios add only 4% and 35% to the global 2020 timber C stocks in buildings by 2050, resulting in timber C stocks in buildings of 8 GtC and 12 GtC, respectively (Figure 2C). By contrast, the two scenarios with a higher wood share in construction (*Floorspace*^*+*^*_timber*^*+*^ and *Floorspace*^*-*^_*_*_*timber*^*+*^*_cascade*^*+*^) show a strong increase in industrial roundwood demand. In these scenarios, despite stagnation and decline after the late 2030s due to population dynamics, industrial roundwood demand in 2050 reaches values of 1900 MtCyr^−1^ and 930 MtCyr^−1^, exceeding the respective 2020 values by factors 1.8 and 1.5 (Figure 2A). Timber stocks in buildings in these two scenarios reach 31 and 19 GtC, respectively, by 2050. Highest demand and accumulation rates are projected in Asia (China, India, other Asian countries (ASIA_Oth)) and Sub-Saharan Africa (SSA), with variations across scenarios persisting at the regional scale (Figures 2B and 2D, Extended Data Figures 1 and 2). In China, the variation across scenarios is highest, which is a result of the huge unused timber construction potential at relatively high per-capita stock levels, combined with a relatively short building lifetime.

The 2020 values in those RECC model scenarios assuming present-day timber shares in buildings are consistent with other estimates: Global assessments of structural timber demand for 2010 (wood and wood products, incl. posts and poles)^65^ and 2020 (residential, non-residential buildings and civil engineering)^66^ report values of 193 and 182.5 MtCyr^−1^, respectively, compared to 141 (*Floorspace*^*+*^_*_*_*timber*^*∼*^) and 250 MtCyr^−1^ (*Floorspace*^*-*^_*_*_*timber*^*∼*^). Scenario results for *Floorspace*^*+*^_*_*_*timber*^*∼*^ fit well with results by Johnston and Radeloff^20^ for 2020–2050, but lie above values reported by Mishra et al.^21^ for final consumption of wood products in the same period. The high variability across options represented in the socioeconomic scenarios results, inter alia, from different assumptions on per-capita floorspace (values vary across scenarios by factors up to 2 in residential and 1.7 in non-residential buildings, Table S2), pointing to the large lever of demand-side changes in resource use.^67^ This is also in line with Daigneault et al.^37^ who find that roundwood harvest varies more across socio-economic scenarios (SSPs) than across climate policy scenarios.

A sensitivity analysis (see supplemental methods, Extended Data Figure 3) reveals that the pattern of the RECC model results is not affected by variations in assumptions on population dynamics and individual material circularity measures. The model output for industrial roundwood demand is most sensitive to assumptions on roundwood-to-timber yield, and values for construction timber C stocks are most sensitive to assumptions on changes in cascadic use, because of its effects on outflows from the wood use cascade. The sensitivity assessment thus confirms that the four chosen scenarios represent “corner scenarios” suitable for defining the demand-side of an option space.

### Wood supply that meets ecosystem services

**Figure 3:**
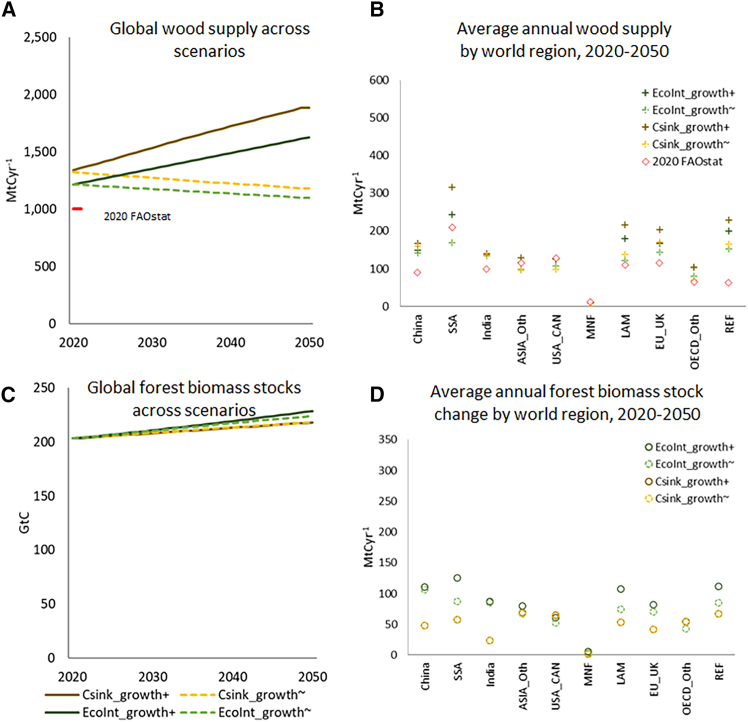
Projections of wood supply and forest biomass stocks without trespassing ecological thresholds in the four scenarios ecological integrity with improved tree growth (EcoInt_growth^+^), ecological integrity without improved tree growth (EcoInt_growth^∼^), forest carbon sink with improved tree growth (Csink_growth^+^) and forest carbon sink without improved tree growth (Csink_growth^∼^) (A) annual global wood supply across scenarios and 2020 value as derived from FAOstat; (B) average annual wood supply in 2020–2050 per world region across scenarios, and 2020 value provided by FAOstat. For a list of the world regions aggregated, see Table S1 and data supplement; (C) global C stocks in forest biomass across scenarios; (D) average annual C stock change in forest biomass per world region across scenarios in 2020–2050. For a list of the world regions aggregated, see Table S1 and data supplement.

According to the CRAFT model results, wood supply from current forest areas within the defined ecological sustainability constraints ranges between 1,100 MtCyr^−1^ (*EcoInt_growth*^*∼*^) and 1,900 MtCyr^−1^ (Csink_growth^+^) in 2050 (Figure 3A), compared to 1,000 MtCyr^−1^ of total wood extracted today, according to FAOstat. Compared to the sustainable allowance modeled for 2020 with CRAFT (1,200 to 1,300 MtCvr^−1^), the projected trends represent a decrease (factor 0.9) or increase (factor 1.4) of global wood harvest. Our wood supply projections are in line with projections of fairly stable global wood harvest in the 21^st^ century in SSP1 and SSP2 scenarios derived in global forest sector models^37^^,^^68^: Daigneault et al.^37^ find that global wood harvest change between 2025 and 2055 will range between factors 0.94 to 1.64 in shared socioeconomic pathways (SSP) scenarios 1 and 2, with scenarios of higher harvest increase also relying on forest expansion by up to 11% in the same period.

According to our simulation, SSA, Latin America (LAM), and the reforming economies of Eastern Europe and the former Soviet Union (REF) have the highest potentials for wood provision. The potentials for wood supply in the forests of SSA and LAM are also most variable across scenarios, and in SSA, ASIA_Oth, USA_CAN, and MNF, some of the scenarios project average annual wood supply in 2020–2050 below the 2020 wood harvest level (Figure 3B, Extended Data Figure 4). All scenarios, as defined in our simulation, forecast an increase in global forest biomass stocks by 2050 to between 218 GtC (*Csink_growth*^*+*^ and *Csink_growth*^*∼*^ scenarios, which are both constrained to reach this stock) and 229 GtC (*EcoInt_growth*^*+*^) in 2050, i.e., 7%–12% above the 2020 value (Figure 3C). Annual forest biomass sinks diverge most across scenarios in SSA, China, and India (Figure 3D, Extended Data Figure 5). Particularly in China and India, the *EcoInt* scenario variations result in strong increase in biomass stocks. While these are rather progressive estimates, the resulting values of biomass density (biomass per unit of forest area) at 56 and 89 tCha^−1^, respectively, remain within the range of default values provided in IPCC guidelines for Asian forests which are between 9 and 179 tCha^−1^, depending on the climate zone and forest type (Table 4.12 in ref.^69^).

The factor most strongly affecting differences in wood supply across scenarios, as well as data sensitivity (Extended Data Figure 6), is the forest growth rate parameter r (discerning the “*_growth*^*+*^” from the “*_growth*^*∼*^” scenarios, see STAR Methods section and supplemental methods for descriptions of model and sensitivity analysis). This corroborates that management, such as planting more rapidly growing trees, and/or changes in environmental conditions can crucially affect annual wood availability, with the potential to enable simultaneous increases in harvest and forest biomass stocks at the global level in the next decades.^37^ Disentangling the effects of forest management vs. environmental conditions on forest growth is thus imperative to better understand possible futures of forest growth.^70^ By contrast, forest biomass C stock accumulation differs much less across scenarios and in the sensitivity assessment. Forest biomass stocks in our scenarios increase only by a fraction of the potential additional forest C storage quantified by Walker et al.^28^ (13%–23%) and Roebroek et al.^29^ (17%–29%), highlighting the impact of wood harvest on forest C accumulation, as well as the long duration of forest recovery.

### Mapping the option space for sustainable wood use

**Figure 4:**
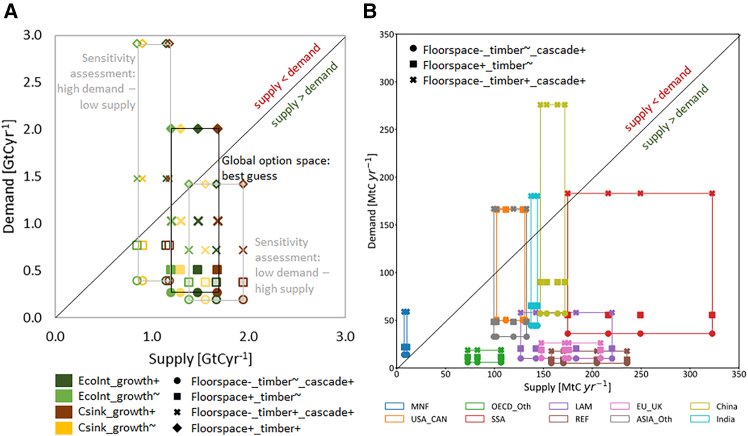
The option space of sustainable wood use, annual average fluxes of sustainable wood supply and demand 2020–2050 (A) Global level wood supply and demand from the building sector, lines connecting the dots indicate the best guess estimate (dark) and the two most extreme variants of the sensitivity assessment: high demand—low supply, and low demand—high supply, respectively. Supply < demand (dots above the diagonal) indicates infeasibility. (B) Global level wood supply and demand by world regions, including only scenario combinations that are globally feasible, lines connecting the dots indicate scenario combinations of one world region. Feasible scenario combinations are those in which supply > demand (dots below the diagonal). Supply < demand (dots above the diagonal) indicates import-dependence at the level of world regions. For a list of the world regions aggregated, see Table S1 and data supplement.

When juxtaposing the supply and demand scenarios at the global scale, 12 out of the 16 scenario combinations meet the global industrial roundwood demand in buildings while respecting ecological limits (Figure 4A). Those scenario combinations where high floorspace provision coincides with increased timber use in buildings (*Floorspace*^*+*^*_timber*^*+*^) are infeasible: industrial roundwood demand clearly exceeds annual wood supply by 0.3–0.8 GtCyr^−1^ across the time period (values displayed above the diagonal line in the panel of Figure 4A). Even when applying the highest values for wood supply and the lowest for demand as computed in our sensitivity assessment (Extended Data Figures 3 and 6), only three of the four *Floorspace*^*+*^*_timber*^*+*^ scenario combinations are feasible (Figure 4A).

In feasible scenario combinations, the primary wood surplus beyond the building sector (i.e., the distance between the diagonal and the dots below the diagonal in Figure 4A) depends more on the share of wood used in construction than on the ecological sustainability constraint applied or the expected future forest growth. Primary wood supply to applications beyond buildings is highest in scenario combinations with low floorspace provision and constant timber use in buildings (*Floorspace*^*-*^*_timber*^*∼*^*_cascade*^*+*^: 0.9–1.4 GtCyr^−1^). Conversely, primary wood supply beyond buildings is critically low when timber in construction is increased in the low-floorspace scenario (*Floorspace*^*-*^*_timber*^*+*^*_cascade*^*+*^: 0.2–0.6 GtCyr^−1^), amounting to only 15%–26% of total wood harvest. This is well below current shares—where 50% of primary wood removals are used for wood fuel,^11^ and c. 30% of the remaining primary industrial roundwood is used in the pulp and paper industry.^65^ Critically, the sensitivity analysis reveals that any scenario combinations that increase timber in construction become infeasible if the highest assumptions on wood demand are applied together with the lowest assumptions on wood supply (Figure 4A).

All scenario combinations that are feasible at the global level depend on some amount of international trade across world regions (Figure 4B): Middle East and Northern Africa (MNF), due to the limited forest resources in the region, is import-dependent across all scenario combinations. China, SSA, India, ASIA_Oth, as well as USA and Canada (USA_CAN) rely on imports in those scenario combinations where timber in construction is increased (*Floorspace*^*-*^*_timber*^*+*^*_cascade*^*+*^). By contrast, forests in LAM, Europe (EU_UK), other OECD countries (OECD_Oth) and REF provide more wood than the respective regional demand across all scenario combinations. SSA is the region with the greatest divergence across scenario combinations, with high variations in both the supply and the demand scenarios. The *Floorspace*^*-*^*_timber*^*∼*^*_cascade*^*+*^ scenario enables self-sufficiency in most regions across the sustainability constraints applied.

**Figure 5:**
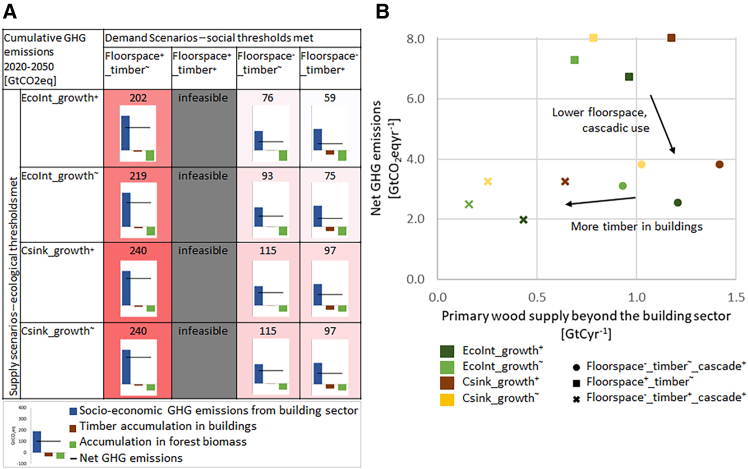
The climate impacts and primary wood supply beyond the building sector across scenario combinations (A) Climate impacts expressed in GtCO_2_eq global cumulative flows in 2020–2050 for each combination of demand (columns) and supply (rows) scenarios. For all feasible scenario combinations (i.e., wood supply exceeds wood demand), values in cells refer to the net effect of socio-economic GHG emissions from the building sector including its energy and material supply, timber accumulation in buildings, and accumulation of forest biomass. In feasible scenarios, darker (red) cells indicate higher net GHG emissions to the atmosphere, while lighter (white) cells conversely indicate lower net GHG emissions. All bar charts are scaled to the same size, numerical values are provided in the data supplement. (B) Net GHG emissions vs. primary wood supply beyond the building sector for all feasible scenario combinations, annual averages 2020–2050.

The climate impact of globally feasible scenario combinations depends not only on the accumulation of C in forests and buildings, but more importantly on the socio-economic scope 1-2-3 GHG emissions associated with the building sector (Figure 5A). In all scenario combinations, and despite the strong C sink in all wood supply scenarios, emissions from the building sector exceed C accumulation in forest biomass and buildings, albeit with strong variations. Across the period 2020–2050, the range in GHG effects of the *Floorspace*^*-*^*_timber*^*+*^*_cascade*^*+*^ scenario combinations (59–97 GtCO_2_eq) amounts to less than half of that of the SSP2 combinations (202–240 GtCO_2_eq). Among the scenario combinations with low floorspace provision, there is a trade-off between GHG emissions and primary wood supply beyond the building sector (Figure 5B): those scenario combinations with the lowest GHG impacts (*Floorspace-_timber+_cascade+*) also have critically little primary wood surplus available to other sectors (0.2–0.6 GtCyr^−1^), less even than the primary wood used beyond the building sector in 2010 (0.8 MtCyr^−1^).^65^ On the other hand, lowering service provision levels while increasing cascadic use reduces the net GHG impact while increasing wood availability for other sectors.

## Discussion

Introducing the option space for sustainable wood use has enabled us to (1) explore which levels of industrial roundwood demand for socially sustainable housing are compatible with ecologically sustainable levels of wood provision from current forest areas, and to (2) assess the respective climate impacts and primary wood availabilities beyond the building sector. In contrast to other forest models assessing trends of timber harvest and forest growth under future carbon price developments,^37^^,^^38^ our approach departs from material and ecosystem service provision as strong sustainability thresholds. It thus allows for incorporating sufficiency options^58^^,^^71^ in the production and consumption of wood (i.e., using less wood where possible), as well as critical ecosystem service provision beyond wood supply.^72^ The results of our analysis do not show likely future trends or optimal production levels. Instead, they provide an orientation as to which combinations of social and ecological sustainability targets can, in principle, be reconciled, and which socio-economic and ecological factors affect the feasibility of scenario combinations the most.

Our results show that different options are available to meet material services in buildings from existing forests by 2050 while respecting ecological limits of timber provision. However, all result in net GHG emissions to the atmosphere from 59 to 240 GtCO_2_eq for 2020–2050. Variability across scenario combinations is shaped more by socio-economic dynamics of wood demand than by ecological variation across wood supply scenarios. Among socio-economic scenarios, the *Floorspace*^*-*^*_timber*^*∼*^*_cascade*^*+*^ scenario results in low net GHG emissions and reduces construction wood demand, leaving more supply for other wood uses while minimizing climate impacts. By contrast, increasing the share of timber in construction under ecological sustainability constraints is only feasible in a low floorspace scenario with higher circularity, and the sensitivity analysis reveals that the feasibility of these scenario combinations is not robust.

An important policy-relevant insight deriving from our research is that compared to potential demand, wood is a scarce resource globally, and ample wood supply to provide for high levels of resource use will not be possible from current forest areas. Therefore, future wood use policies should take into account the ecological constraints in wood supply to avoid national or international forest overuse. Curbing floorspace growth appears as a prerequisite for increasing timber in construction, if ecological forest integrity is to be safeguarded. Within these ecological limits, our findings point to the need for policies to prioritize the use of primary wood toward high-value long-lived products with high displacement of carbon-intensive construction materials, such as long-lived structural timber. Beyond this, our results substantiate calls for incentivizing reuse and cascadic uses of byproducts and postconsumer material flows for those applications with lower quality requirements, such as packaging, containers, or cardboard, while wood bioenergy minimizing and substituted by non-carbon energy as much as possible.^73^^,^^74^

To further increase the usefulness of option space approaches for decision making, the option space approach presented here can be complemented by consequential analyses. Such analyses compare the relative climate impact of two options of wood use by quantifying carbon opportunity costs on both the forest and the built environment sides.^12^ Examples for consequential indicators on the comparative climate impact include the “required displacement factor”,^75^ and the “carbon storage balance in forests”.^76^ A comparison of alternatives within the option space can then identify which combined forest management and wood use strategies are more beneficial to the climate by taking into account the system-wide implications of foregoing either wood harvest or wood use.

In the complex academic and political debate around sustainable wood use, the option space we introduce here serves as an easily communicable boundary concept to facilitate dialogue between researchers from different disciplines, as well as between academia and policymakers. The concept is sufficiently complex to represent the intricacies of social and ecological sustainability challenges connected to wood use, but enables to overcome stock-flow-failures^77^ or single-minded perspectives focusing, e.g., on substitution effects or nature conservation alone. We thus lay the ground for nuanced debates and deliberations of sustainable wood use.

### Limitations of the study

As a first operationalization, the model coupling presented here relies on some rather coarse assumptions, and the sensitivity analysis of our results displays low robustness with regards to the feasibility of increasing timber use in construction. Future research will need to advance models to narrow down these uncertainties. Beyond that, the option space approach offers high potential for further analyses, including the coverage more diverse sustainability dimensions, i.e., more material, energy, and ecosystem services. The functional resolution currently includes only one wood use category (structural timber) and thus provides only a proxy for total wood demand in buildings. Investigating the full spectrum of wood quality grades and multiple end uses can lead to a more complete picture of the social and economic benefits of wood use and the substitution potential for fossil-fuel-based energy carriers and materials. Furthermore, analyses at multiple time horizons merit future attention, explicitly addressing the dynamic long-term interplay of ecological (e.g., climate impacts, disturbance regimes) and socio-economic trends (e.g., decarbonization of the economy, technological change).

## Resource availability

### Lead contact

Requests for further information and resources should be directed to and will be fulfilled by the lead contact, Simone Gingrich (simone.gingrich@boku.ac.at).

### Materials availability

This study did not generate new unique reagents.

### Data and code availability

• All data presented in this study have been deposited at Zenodo and are publicly available at https://zenodo.org/records/16870421.
• Code for the RECC model is available here https://github.com/IndEcol/RECC-ODYM.
• Methods details are provided in Document S2: supplemental methods. RECC model details are published and continuously updated at https://www.industrialecology.uni-freiburg.de/odym-recc. CRAFT model details are introduced in Le Noë et al. (2020)^59^ and elaborated for the global scale in Le Noë et al. (2021).^60^ Any additional information required to further analyze the data reported in this paper is available from the lead contact upon request.

## Acknowledgments

10.13039/501100007601European Union’s Horizon 2020 research and innovation program—10.13039/501100000781European Research Council (HEFT grant agreement no. 757995, MAT_STOCKS’, grant agreement no. 741950), Austrian Climate Resarch Panel (Unravel KR20AC0K18081), European Union's Horizon Europe programme (CircEUlar, grant agreement no 101056810), and 10.13039/501100002428Austrian Science Fund (FWF, project REMASS, doi: 10.55776/EFP5). S.P. acknowledges funding from the CIRCOMOD project, funded by the Horizon Program of the European Union under grant agreement no. 101056868.

## Author contributions

Conceptualization, investigation, writing – original draft, visualization: S.G. conceptualization, formal analysis, visualization, writing – review & editing: S.M. conceptualization, writing – review & editing: K.-H.E. conceptualization, writing – review & editing: H.H. conceptualization, writing – review & editing: J.L.N. conceptualization, investigation, visualization, writing – review & editing: L.K. conceptualization, investigation, writing – review & editing: A.M. conceptualization, writing – review & editing: A.S. conceptualization, visualization, writing – review & editing: D.W. conceptualization, formal analysis, writing – review & editing: S.P.

## Declaration of interests

The authors declare no competing interests.

## STAR★Methods

### Key resources table

**Table:** 

REAGENT or RESOURCE	SOURCE	IDENTIFIER
**Deposited data**		

RECC model results and results of sensitivity analysis	This paper	https://zenodo.org/uploads/16870421
CRAFT model results and results of sensitivity analysis	This paper	https://zenodo.org/uploads/16870421
RECC v2.5 global building stock model input and result database	Pauliuk et al. (2020)^56^	https://zenodo.org/records/12752350

**Software and algorithms**		

RECC Python model code	Pauliuk & Heeren (2020)^78^	https://github.com/IndEcol/RECC-ODYM
RECC v2.5 model documentation	Pauliuk (2023)^79^	https://doi.org/10.6094/UNIFR/242061
CRAFT model description	Le Noë (2020)^59^	https://doi.org/10.1111/gcb.15004
CRAFT global model application and input data for this study	Le Noë (2021)^60^	https://doi.org/10.1038/s41467-021-26398-2

### Method details

We adopt an integrated stock-flow perspective to quantify the C dynamics associated with wood use in the global building sector (Figure S1). This perspective is the foundation for consistently coupling the model outputs from the RECC and CRAFT models on (i) the socio-economic demand for industrial roundwood to provide housing services under different socio-economic scenarios and (ii) the allowable timber harvest that can be provided from current forest areas under specific ecological constraints, covering the period 2020-2050 at the global and world regional levels. Model coupling thus allows to assess the feasibility of a broad range of future demand and supply scenario combinations that meet social and ecological sustainability criteria, respectively.

Beyond quantifying whether specific combinations of supply and demand are feasible, model coupling enables quantifying the primary wood supply to sectors beyond buildings (i.e., the difference between supply and building sector demand), and the overall climate impact of the scenario combination (i.e., the building sector emissions minus C accumulation in forests and buildings). The models combined are unique in that they depict the construction wood stock dynamics in buildings in great detail and consistency (RECC) and provide a realistic corridor of forest carbon pools and harvesting potentials (CRAFT) at the global scale.

### Quantification and statistical analysis

#### RECC scenarios on timber demand in buildings

We use the RECC^56^^,^^80^ model of the energy service cascade and the stock-flow-service nexus for cement, steel, and wood in the global building stock to quantify socio-economic wood use to meet global housing services under different socio-economic scenarios (Figure S2).^56^ We quantify the demand for structural timber in residential and non-residential buildings (*f*_*I*_ in Figure S2), changes in socio-economic timber stocks in buildings (ΔB,w in Figure S2), cascadic use of wood (*f*_*V*_ in Figure S2), and CO_2_ emissions from cement and fossil energy use in the building sector and of upstream inputs for building materials (*f*_*VI*_ in Figure S2). For each flow and stock, we quantify annual values at the level of ten world regions (Table S1, extended data file; Figure S3).

The RECC model uses expert-defined per-capita m^2^ trajectories for residential and non-residential buildings based on the storylines of the shared socioeconomic pathways (SSP). A stock-driven model^81^ determines future construction to replace and expand the in-use stock of buildings. Building archetypes with different degrees of material and energy efficiency (52 residential and 96 non-residential building types) are scaled up to link demand for new floorspace to the different construction materials, including structural timber. RECC computes in-use stocks of timber in buildings and material and energy supply to maintain, expand, and operate the global building stock (e.g., annual inflow of construction wood, annual energy demand of construction), as well as a cascading option for end-of-life structural timber and the greenhouse gas emissions for energy and material supply for different decarbonization scenarios. To estimate future GHG emissions of the building sector (scope 1, 2, and 3), building renovation, electrification, and low-carbon electricity supply are considered, as well as substantial reductions of GHG emissions in steel production (via hydrogen as reduction agent) and in cement (via clinker substitution). RECC operates at annual intervals and at the spatial resolution of ten world regions.

For the quantification of wood demand in the building sector, we adapted four previously-published socio-economic scenarios^55^ (Table S3) that cover the range of possible future dynamics in industrial roundwood demand for buildings. To this end, we modify three parameters: (1) the overall service level of buildings, approximated by the 2050 target values for m^2^ per-capita of building area (Table S2), (2) the share of wood-intensive buildings in new construction needed to meet the respective service levels, and (3) the level of circularity or cascadic use of wood (see Table “Key characteristics of scenarios for wood demand in the building sector as quantified in the RECC model”). As for (1), we discern (a) a business as usual scenario (*Floorspace*^*+*^), building on the storyline of the shared socioeconomic pathways “middle-of the road” scenario (SSP2^57^), with increasing per-capita floorspace, and (b) a low energy and material demand (LEMD^58^) scenario (*Floorspace*^*-*^) that implies a lower per-capita floorspace than SSP2 and upscales the most material and energy efficient archetypes in construction, resulting in lower overall resource demand to provide material services. For (2), we implement (a) a scenario of constant (*_timber*^*∼*^) and (b) increased wood use per m^2^ (*_timber*^*+*^), assuming different sets of construction technologies. By combining these two dimensions, we arrive at four distinct scenarios. To display the full option space of wood demand, we additionally assume (3) that the two *Floorspace*^*+*^ scenarios (i.e., those with higher material demand) retain the current design, manufacturing, lifetime, and recycling practices, while the two *Floorspace*^*-*^ scenarios additionally increase the circularity, i.e. both the re-use rate and the fraction of cascadic use: The higher re-use rate of end-of life structural timber (14%) is based on the assumption that the theoretical potential of reusing timber is similar to the identified reuse potential of steel or concrete, but, due to quality degradation, only half of the theoretically available timber suitable for reuse can be reused in practice, provided that supporting regulations and/or economic incentives are in place (*_cascade*^*+*^). For a detailed presentation of the circularity assumptions in the RECC model, see Pauliuk et al.^55^

**Table:** Key characteristics of scenarios for wood demand in the building sector as quantified in the RECC model

Demand scenarios	Socio-economic service levels	Wood intensity in new buildings	Circularity of construction sector	Structural timber yield from industrial roundwood
*Floorspace*^*+*^*_timber*^*∼*^	33-84 m^2^/cap (residential), 12-30 m^2^/cap (non-residential)	0-45 kg timber per m^2^, depending on building type	Low: no re-use of end-of-life structural timber, 0-30% is cascaded (another 25 yr lifetime in form of other products)	50% (34-60%, depending on world region)
*Floorspace*^*+*^*_timber*^*+*^	33-84 m^2^/cap (residential), 12-30 m^2^/cap (non-residential)	100-260 kg timber per m^2^, depending on building type	Low: no re-use of end-of-life structural timber, 0-30% is cascaded (another 25 yr lifetime in form of other products)	50% (34-60%, depending on world region)
*Floorspace*^*-*^*_timber*^*∼*^*_cascade*^*+*^	19-43 m^2^/cap (residential), 7-18 m^2^/cap (non-residential)	0-45 kg timber per m^2^, depending on building type	High: 14% re-use of end-of-life structural timber, 50% is cascaded (another 25 yr lifetime in form of other products)	50% (34-60%, depending on world region)
*Floorspace*^*-*^*_timber*^*+*^*_cascade*^*+*^	19-43 m^2^/cap (residential), 7-18 m^2^/cap (non-residential)	100-260 kg timber per m^2^, depending on building type	High: 14% re-use of end-of-life structural timber, 50% is cascaded (another 25 yr lifetime in form of other products)	50% (34-60%, depending on world region)

The scenarios differ in terms of socio-economic service levels, wood intensity in new buildings, and circularity of the construction sector. They do not differ in structural timber yields.

Beyond displaying the range of options for wood demand to meet housing services through these scenarios, we conduct a sensitivity analysis to identify how sensitive the RECC model output is to modifications in assumptions on population, timber yields in sawmills, and circularity in resource use (Table S4). For more detail on the RECC model application for this study, see the supplemental methods.

#### CRAFT scenarios on timber harvest supply

We use the CRAFT^59^^,^^60^ model to quantify wood harvest potentials and forest biomass C stock dynamics in four independent scenarios for 2020-2050. The CRAFT^59^^,^^60^ model is a parsimonious stock-flow forest model that uses national-level input data on trends in forest area, forest biomass stocks and wood harvest, derived from FAOstat for 1990-2020 to establish a dynamic relationship between gross annual increment and forest biomass stocks at the national level:

(Equation 1) $${GAI}_{c,t}={r_{c,t}B}_{c,t}\left(1-\frac{B_{c,t}}{K_{c}}\right)$$

With *GAI* denoting gross annual increment (tCha^-1^yr^-1^); *r* the forest growth rate parameter (yr^-1^); *B* the forest biomass density (tCha^-1^), and *K* the theoretical carrying capacity (tCha^-1^). Subscripts _*c*_ and _*t*_ indicate country- and year-specific values respectively. Based on the procedure developed in Le Noë et al.^60^ and modified for this study as described in the supplemental methods, CRAFT optimizes the variables *r*, *K*, and *α* (the linear growth rate of *r*_*c*_ between 1990 and 2020) such that national biomass stocks are accurately reproduced in the period 1990-2020.

We use the variables *r*, *K*, and *α* to develop four scenarios forecasting wood harvest and forest biomass stock trends from 2020-2050 at country level, starting from biomass stocks and forest areas in 2020. All scenarios assume constant 2020 forest areas, but vary in terms of two levers that are independently modified (Table “Key characteristics of wood supply scenarios quantified in the CRAFT model”): (1) the ecological limits considered, implemented as constraints to harvest and biomass stock trends respectively, and (2) the dynamics of future forest growth assumed, implemented as different trends in *α* and the forest growth rate parameter *r*.

(1) The two ecological limits correspond to the two core planetary boundaries, i.e. biosphere integrity and climate change,^82^ and are expressed in the scenario abbreviations starting with *EcoInt* for “ecological integrity” and *Csink* for “forest C sink”, respectively. For ecological integrity, we define a target limiting management intensity, a major driver of forest biodiversity loss,^61^ such that annual wood extraction equals no more than 75% of gross annual increment at the national level. This is a conservative limit, for comparison annual fellings in the European Union averaged between 75% and 85% of net annual increment in 2015.^83^ Though arguably debatable in the precise number applied, this limit acknowledges that the more biomass is extracted from an ecosystem for societal use, the less remains for all other species to thrive.^84^ The other ecological limit implemented refers to the need for restoring forest biomass stocks, to maximize C sinks in forests and mitigate climate change. Values for feasible forest C sinks were derived from country-level technical potentials for increasing C sequestration in forests through improved management, such as reduced-impact logging, non-intensive wood production and extended harvest cycles.^36^ Using the CRAFT model, we quantified harvest levels that are compatible with fulfilling the suggested potentials for forest C sinks.
(2) The two assumptions on future forest growth correspond to “best” and “worst case” scenarios, expecting increasing future forest growth and no increase, respectively (scenario abbreviations ending in *_growth*^*+*^ or _*growth*^*∼*^). The “best case” scenarios (*_growth*^*growth+*^) assume continuously increasing country-level forest growth rate parameters *r* in 2020-2050 (and a stabilization in those countries where *r* declined in 1990-2020). Such increased growth rates may result from vegetation greening^62^ due to environmental change, and/or from socio-economic interventions, such as expansion of plantations into existing forest areas, or changing spatial forest patterns even if total forest area remains constant. In the “worst case” scenarios (_*growth*^*∼*^), we assume that vegetation browning^63^ and/or increased disturbances due to future climate impacts^64^ will lead to stable growth rates in countries where forest growth rates grew in 1990-2020 and to continuously declining growth rates in countries where it already declined in 1990-2020 (see Figure S3 and data supplement for trends in growth parameters at national levels).

**Table:** Key characteristics of wood supply scenarios quantified in the CRAFT model

Scenario	Ecological sustainability target to be safeguarded	Forest growth dynamics expected
*EcoInt_growth*^*+*^	Annual harvest = 75% of gross annual increment (GAI). Country-level GAI computed in CRAFT.	Country-level increase in forest growth continues, decline in forest growth ends. If α_c,1990-2020_>1, then α_c,2020-2050_= α_c,1990-2020_; if α_c,1990-2020_<1, then α_c,2020-2050_=1 (α_c,1990-2020_ derived from CRAFT optimisation)
*EcoInt_growth*^*∼*^	Annual harvest = 75% of gross annual increment (GAI). Country-level GAI computed in CRAFT.	Country-level decline in forest growth continues, increase in forest growth ends. If α_c,1990-2020_<1, then α_c,2020-2050_= α_c,1990-2020_; if α_c,1990-2020_>1, then α_c,2020-2050_=1 (α_c,1990-2020_ derived from CRAFT optimisation)
*Csink_growth*^*+*^	Country-level forest C-sink equals technical potential for C-sink through improved forest management reported in Roe et al.^36^	Country-level increase in forest growth continues, decline in forest growth ends. If α_c,1990-2020_>1, then α_c,2020-2050_= α_c,1990-2020_; if α_c,1990-2020_<1, then α_c,2020-2050_=1 (α_c,1990-2020_ derived from CRAFT optimisation)
*Csink_growth*^*∼*^	Country-level forest C-sink equals technical potential for C-sink through improved forest management reported in Roe et al.^36^	Country-level decline in forest growth continues, increase in forest growth ends. If α_c,1990-2020_<1, then α_c,2020-2050_= α_c,1990-2020_; if α_c,1990-2020_>1, then α_c,2020-2050_=1 (α_c,1990-2020_ derived from CRAFT optimisation)

We assess the sensitivity of CRAFT model results, building on information on data reliability provided the global Forest Resource Assessment^11^ (Table S5), by modifying three model input parameters (Table S6), i.e., biomass density in 2020, country-level forest growth rates, and country-level changes in forest growth rates.

#### Model coupling

For each combination of scenarios (4 x 4 scenarios resulting in 16 scenario combinations), we assess whether cumulative wood supply in the period 2020-2050 exceeds industrial roundwood demand for structural timber used in the building sector. If supply exceeds demand, we consider a scenario combination as feasible. For the purpose of model coupling, wood flows are quantified as total wood harvest in MtCyr^-1^, excluding bark. We assume that any wood harvest can be used as material input to the building sector in some form, with the conversion rates from roundwood to structural timber of 30% for hardwood and 70% for softwood, ignoring any additional quality criteria of wood in construction that commonly lead to complex wood processing chains.^65^^,^^85^ We do this both at the level of world regions (with international trade *within* each of the ten world regions defined in the RECC model (Table S1)) and globally (with international trade *between* the world regions, allowing for regional deficits in wood supply to be compensated by surplus in other regions).

For globally feasible scenario combinations, we quantify two indicators: First, the overall GHG impact of the building sector is assessed by subtracting the C accumulation in buildings and forest biomass from socio-economic GHG emissions in the building sector (i.e., emissions from fossil fuel use and cement production). This indicator allows for evaluating to which extent forest C sinks are overcompensated by emissions to the atmosphere from the building sector. Secondly, we also quantify the amount of primary wood surplus available beyond the building sector by subtracting, for each scenario combination, industrial roundwood demand for housing services provision from wood supply. This indicator informs about the extent to which the building sector consumes available wood harvest and conversely, how much primary wood is available for other material or energy use of wood. The specific use of primary wood surplus, or its indirect climate impacts via substitution, are not further quantified.

## Supplemental information

Document S1. Extended Data Figures 1–6

## References

[1] Griscom B.W., Busch J., Cook-Patton S.C., Ellis P.W., Funk J., Leavitt S.M., Lomax G., Turner W.R., Chapman M., Engelmann J. (2020). National mitigation potential from natural climate solutions in the tropics. Philos. Trans. R. Soc. B.

[2] Roe S., Streck C., Obersteiner M., Frank S., Griscom B., Drouet L., Fricko O., Gusti M., Harris N., Hasegawa T. (2019). Contribution of the land sector to a 1.5 °C world. Nat. Clim. Chang..

[3] IPCC (2023). Climate Change 2022 - Mitigation of Climate Change.

[4] Houghton R.A., Nassikas A.A. (2018). Negative emissions from stopping deforestation and forest degradation, globally. Glob. Chang. Biol..

[5] Rudel T.K., Meyfroidt P., Chazdon R., Bongers F., Sloan S., Grau H.R., Van Holt T., Schneider L. (2020). Whither the forest transition? Climate change, policy responses, and redistributed forests in the twenty-first century. Ambio.

[6] Erb K., Haberl H., Le Noë J., Tappeiner U., Tasser E., Gingrich S. (2022). Changes in perspective needed to forge ‘no-regret’ forest-based climate change mitigation strategies. GCB Bioenergy.

[7] Cowie A.L., Berndes G., Bentsen N.S., Brandão M., Cherubini F., Egnell G., George B., Gustavsson L., Hanewinkel M., Harris Z.M. (2021). Applying a science-based systems perspective to dispel misconceptions about climate effects of forest bioenergy. GCB Bioenergy.

[8] Searchinger T.D., Wirsenius S., Beringer T., Dumas P. (2018). Assessing the efficiency of changes in land use for mitigating climate change. Nature.

[9] Mather-Gratton Z.J., Larsen S., Bentsen N.S. (2021). Understanding the sustainability debate on forest biomass for energy in Europe: A discourse analysis. PLoS One.

[10] Kalt G., Mayer A., Theurl M.C., Lauk C., Erb K.H., Haberl H. (2019). Natural climate solutions versus bioenergy: Can carbon benefits of natural succession compete with bioenergy from short rotation coppice?. GCB Bioenergy.

[11] FAO (2020).

[12] Soimakallio S., Kalliokoski T., Lehtonen A., Salminen O. (2021). On the trade-offs and synergies between forest carbon sequestration and substitution. Mitig. Adapt. Strateg. Glob. Chang..

[13] Skytt T., Englund G., Jonsson B.-G. (2021). Climate mitigation forestry—temporal trade-offs. Environ. Res. Lett..

[14] Maierhofer D., Van Karsbergen V., Potrč Obrecht T., Ruschi Mendes Saade M., Gingrich S., Streicher W., Erb K.-H., Passer A. (2024). Linking forest carbon opportunity costs and greenhouse gas emission substitution effects of wooden buildings: The climate optimum concept. Sustain. Prod. Consum..

[15] Geng A., Yang H., Chen J., Hong Y. (2017). Review of carbon storage function of harvested wood products and the potential of wood substitution in greenhouse gas mitigation. For. Pol. Econ..

[16] Kaufmann L., Wiedenhofer D., Cao Z., Theurl M.C., Lauk C., Baumgart A., Gingrich S., Haberl H. (2024). Society’s material stocks as carbon pool: an economy-wide quantification of global carbon stocks from 1900–2015. Environ. Res. Lett..

[17] Watari T., Yamashita N., Serrenho A.C. (2024). Net-Zero Embodied Carbon in Buildings with Today’s Available Technologies. Environ. Sci. Technol..

[18] Bjånesøy S., Kinnunen A., Einarsdóttir H., Heinonen J. (2023). Carbon storage in the built environment: a review. Environ. Res. Infrastruct. Sustain..

[19] Himes A., Busby G. (2020). Wood buildings as a climate solution. Dev. Built Environ..

[20] Johnston C.M.T., Radeloff V.C. (2019). Global mitigation potential of carbon stored in harvested wood products. Proc. Natl. Acad. Sci. USA.

[21] Mishra A., Humpenöder F., Churkina G., Reyer C.P.O., Beier F., Bodirsky B.L., Schellnhuber H.J., Lotze-Campen H., Popp A. (2022). Land use change and carbon emissions of a transformation to timber cities. Nat. Commun..

[22] Duan Z., Huang Q., Zhang Q. (2022). Life cycle assessment of mass timber construction: A review. Build. Environ..

[23] Luyssaert S., Schulze E.-D., Börner A., Knohl A., Hessenmöller D., Law B.E., Ciais P., Grace J. (2008). Old-growth forests as global carbon sinks. Nature.

[24] Rockström J., Beringer T., Hole D., Griscom B., Mascia M.B., Folke C., Creutzig F. (2021). We need biosphere stewardship that protects carbon sinks and builds resilience. Proc. Natl. Acad. Sci. USA.

[25] Bastin J.-F., Finegold Y., Garcia C., Mollicone D., Rezende M., Routh D., Zohner C.M., Crowther T.W. (2019). The global tree restoration potential. Science.

[26] Erb K.-H., Kastner T., Plutzar C., Bais A.L.S., Carvalhais N., Fetzel T., Gingrich S., Haberl H., Lauk C., Niedertscheider M. (2018). Unexpectedly large impact of forest management and grazing on global vegetation biomass. Nature.

[27] Mo L., Zohner C.M., Reich P.B., Liang J., de Miguel S., Nabuurs G.-J., Renner S.S., van den Hoogen J., Araza A., Herold M. (2023). Integrated global assessment of the natural forest carbon potential. Nature.

[28] Walker W.S., Gorelik S.R., Cook-Patton S.C., Baccini A., Farina M.K., Solvik K.K., Ellis P.W., Sanderman J., Houghton R.A., Leavitt S.M. (2022). The global potential for increased storage of carbon on land. Proc. Natl. Acad. Sci. USA.

[29] Roebroek C.T.J., Duveiller G., Seneviratne S.I., Davin E.L., Cescatti A. (2023). Releasing global forests from human management: How much more carbon could be stored?. Science.

[30] Nolan C.J., Field C.B., Mach K.J. (2021). Constraints and enablers for increasing carbon storage in the terrestrial biosphere. Nat. Rev. Earth Environ..

[31] Erb K.-H., Gingrich S. (2022). Biomass—Critical limits to a vital resource. One Earth.

[32] Kaarakka L., Cornett M., Domke G., Ontl T., Dee L.E. (2021). Improved forest management as a natural climate solution: A review. Ecol. Solut. Evid..

[33] Soimakallio S., Böttcher H., Niemi J., Mosley F., Turunen S., Hennenberg K.J., Reise J., Fehrenbach H. (2022). Closing an open balance: The impact of increased tree harvest on forest carbon. GCB Bioenergy.

[34] Law B.E., Hudiburg T.W., Berner L.T., Kent J.J., Buotte P.C., Harmon M.E. (2018). Land use strategies to mitigate climate change in carbon dense temperate forests. Proc. Natl. Acad. Sci. USA.

[35] Aryapratama R., Pauliuk S. (2022). Life cycle carbon emissions of different land conversion and woody biomass utilization scenarios in Indonesia. Sci. Total Environ..

[36] Roe S., Streck C., Beach R., Busch J., Chapman M., Daioglou V., Deppermann A., Doelman J., Emmet-Booth J., Engelmann J. (2021). Land-based measures to mitigate climate change: Potential and feasibility by country. Glob. Chang. Biol..

[37] Daigneault A., Baker J.S., Guo J., Lauri P., Favero A., Forsell N., Johnston C., Ohrel S.B., Sohngen B. (2022). How the future of the global forest sink depends on timber demand, forest management, and carbon policies. Glob. Environ. Change..

[38] Austin K.G., Baker J.S., Sohngen B.L., Wade C.M., Daigneault A., Ohrel S.B., Ragnauth S., Bean A. (2020). The economic costs of planting, preserving, and managing the world’s forests to mitigate climate change. Nat. Commun..

[39] Favero A., Daigneault A., Sohngen B., Baker J. (2023). A system-wide assessment of forest biomass production, markets, and carbon. GCB Bioenergy.

[40] Pomponi F., Hart J., Arehart J.H., D’Amico B. (2020). Buildings as a Global Carbon Sink? A Reality Check on Feasibility Limits. One Earth.

[41] Hertwich E.G., Ali S., Ciacci L., Fishman T., Heeren N., Masanet E., Asghari F.N., Olivetti E., Pauliuk S., Tu Q., Wolfram P. (2019). Material efficiency strategies to reducing greenhouse gas emissions associated with buildings, vehicles, and electronics—a review. Environ. Res. Lett..

[42] Hudiburg T.W., Law B.E., Moomaw W.R., Harmon M.E., Stenzel J.E. (2019). Meeting GHG reduction targets requires accounting for all forest sector emissions. Environ. Res. Lett..

[43] Keith H., Vardon M., Obst C., Young V., Houghton R.A., Mackey B. (2021). Evaluating nature-based solutions for climate mitigation and conservation requires comprehensive carbon accounting. Sci. Total Environ..

[44] Peng L., Searchinger T.D., Zionts J., Waite R. (2023). The carbon costs of global wood harvests. Nature.

[45] Kalt G., Wiedenhofer D., Görg C., Haberl H. (2019). Conceptualizing energy services: A review of energy and well-being along the Energy Service Cascade. Energy Res. Social Sci..

[46] Whiting K., Carmona L.G., Carrasco A. (2022). The resource service cascade: A conceptual framework for the integration of ecosystem, energy and material services. Environ. Dev..

[47] Potschin M.B., Haines-Young R.H. (2011). Ecosystem services: Exploring a geographical perspective. Prog. Phys. Geogr. Earth Environ..

[48] Fu B., Wang S., Su C., Forsius M. (2013). Linking ecosystem processes and ecosystem services. Curr. Opin. Environ. Sustain..

[49] Raworth K. (2017).

[50] Fanning A.L., O’Neill D.W., Hickel J., Roux N. (2021). The social shortfall and ecological overshoot of nations. Nat. Sustain..

[51] O’Neill D.W., Fanning A.L., Lamb W.F., Steinberger J.K. (2018). A good life for all within planetary boundaries. Nat. Sustain..

[52] Barbieri P., Pellerin S., Seufert V., Smith L., Ramankutty N., Nesme T. (2021). Global option space for organic agriculture is delimited by nitrogen availability. Nat. Food.

[53] Erb K.-H., Lauk C., Kastner T., Mayer A., Theurl M.C., Haberl H. (2016). Exploring the biophysical option space for feeding the world without deforestation. Nat. Commun..

[54] Wu F., Pfenninger S., Muller A. (2024). Land-free bioenergy from circular agroecology—a diverse option space and trade-offs. Environ. Res. Lett..

[55] Pauliuk S., Carrer F., Heeren N., Hertwich E.G. (2024). Scenario analysis of supply- and demand-side solutions for circular economy and climate change mitigation in the global building sector. J. Ind. Ecol..

[56] Pauliuk S., Fishman T., Heeren N., Berrill P., Tu Q., Wolfram P., Hertwich E.G. (2021). Linking service provision to material cycles: A new framework for studying the resource efficiency–climate change (RECC) nexus. J. Ind. Ecol..

[57] Riahi K., Van Vuuren D.P., Kriegler E., Edmonds J., O’Neill B.C., Fujimori S., Bauer N., Calvin K., Dellink R., Fricko O. (2017). The Shared Socioeconomic Pathways and their energy, land use, and greenhouse gas emissions implications: An overview. Glob. Environ. Change.

[58] Grubler A., Wilson C., Bento N., Boza-Kiss B., Krey V., McCollum D.L., Rao N.D., Riahi K., Rogelj J., De Stercke S. (2018). A low energy demand scenario for meeting the 1.5 °C target and sustainable development goals without negative emission technologies. Nat. Energy.

[59] Le Noë J., Matej S., Magerl A., Bhan M., Erb K., Gingrich S. (2020). Modeling and empirical validation of long-term carbon sequestration in forests (France, 1850–2015). Glob. Change Biol..

[60] Le Noë J., Erb K.-H., Matej S., Magerl A., Bhan M., Gingrich S. (2021). Altered growth conditions more than reforestation counteracted forest biomass carbon emissions 1990–2020. Nat. Commun..

[61] Oettel J., Lapin K. (2021). Linking forest management and biodiversity indicators to strengthen sustainable forest management in Europe. Ecol. Indic..

[62] Zhang Y., Song C., Band L.E., Sun G., Li J. (2017). Reanalysis of global terrestrial vegetation trends from MODIS products: Browning or greening?. Remote Sens. Environ..

[63] Pan N., Feng X., Fu B., Wang S., Ji F., Pan S. (2018). Increasing global vegetation browning hidden in overall vegetation greening: Insights from time-varying trends. Remote Sens. Environ..

[64] Seidl R., Thom D., Kautz M., Martin-Benito D., Peltoniemi M., Vacchiano G., Wild J., Ascoli D., Petr M., Honkaniemi J. (2017). Forest disturbances under climate change. Nat. Clim. Chang..

[65] Bais A.L.S., Lauk C., Kastner T., Erb K. (2015). Global patterns and trends of wood harvest and use between 1990 and 2010. Ecol. Econ..

[66] Wiedenhofer D., Streeck J., Wieland H., Grammer B., Baumgart A., Plank B., Helbig C., Pauliuk S., Haberl H., Krausmann F. (2024). From extraction to end-uses and waste management: Modeling economy-wide material cycles and stock dynamics around the world. J. Ind. Ecol..

[67] Creutzig F., Roy J., Lamb W.F., Azevedo I.M.L., Bruine de Bruin W., Dalkmann H., Edelenbosch O.Y., Geels F.W., Grubler A., Hepburn C. (2018). Towards demand-side solutions for mitigating climate change. Nat. Clim. Chang..

[68] Lauri P., Forsell N., Gusti M., Korosuo A., Havlík P., Obersteiner M. (2019). Global Woody Biomass Harvest Volumes and Forest Area Use Under Different SSP-RCP Scenarios. J. For. Econ..

[69] IPCC (2019). 2019 Refinement to the 2006 IPCC Guidelines for Greenhouse Gas Inventories. Volume 4. Agriculture, Forestry and Other Land Use.

[70] Keenan T.F., Williams C.A. (2018). The Terrestrial Carbon Sink. Annu. Rev. Environ. Resour..

[71] Sugiyama M., Wilson C., Wiedenhofer D., Boza-Kiss B., Cao T., Chatterjee J.S., Chatterjee S., Hara T., Hayashi A., Ju Y. (2024). High with low: Harnessing the power of demand-side solutions for high wellbeing with low energy and material demand. Joule.

[72] Díaz S., Pascual U., Stenseke M., Martín-López B., Watson R.T., Molnár Z., Hill R., Chan K.M.A., Baste I.A., Brauman K.A. (2018). Assessing nature’s contributions to people. Science.

[73] Sikkema R., Junginger M., McFarlane P., Faaij A. (2013). The GHG contribution of the cascaded use of harvested wood products in comparison with the use of wood for energy—A case study on available forest resources in Canada. Environ. Sci. Pol..

[74] Szichta P., Risse M., Weber-Blaschke G., Richter K. (2022). Potentials for wood cascading: A model for the prediction of the recovery of timber in Germany. Resour. Conserv. Recycl..

[75] Hurmekoski E., Smyth C.E., Stern T., Verkerk P.J., Asada R. (2021). Substitution impacts of wood use at the market level: a systematic review. Environ. Res. Lett..

[76] Fehrenbach H., Bischoff M., Böttcher H., Reise J., Hennenberg K.J. (2022). The Missing Limb: Including Impacts of Biomass Extraction on Forest Carbon Stocks in Greenhouse Gas Balances of Wood Use. Forests.

[77] Cronin M.A., Gonzalez C., Sterman J.D. (2009). Why don’t well-educated adults understand accumulation? A challenge to researchers, educators, and citizens. Organ. Behav. Hum. Decis. Process..

[78] Pauliuk S., Heeren N. (2020). ODYM—An open software framework for studying dynamic material systems: Principles, implementation, and data structures. J. Ind. Ecol..

[79] Pauliuk S. (2023).

[80] Fishman T., Heeren N., Pauliuk S., Berrill P., Tu Q., Wolfram P., Hertwich E.G. (2021). A comprehensive set of global scenarios of housing, mobility, and material efficiency for material cycles and energy systems modeling. J. Ind. Ecol..

[81] B Müller D. (2006). Stock dynamics for forecasting material flows—Case study for housing in The Netherlands. Ecol. Econ..

[82] Richardson K., Steffen W., Lucht W., Bendtsen J., Cornell S.E., Donges J.F., Drüke M., Fetzer I., Bala G., Von Bloh W. (2023). Earth beyond six of nine planetary boundaries. Sci. Adv..

[83] Camia A., Giuntoli J., Jonsson R., Robert N., Cazzaniga N.E., Jasinevicius G., Avitabile V., Grassi G., Barredo J.I., Mubareka S. (2021).

[84] Running S.W. (2012). A Measurable Planetary Boundary for the Biosphere. Science.

[85] Wang R., Haller P. (2024). Dynamic material flow analysis of wood in Germany from 1991 to 2020. Resour. Conserv. Recycl..

