# Supplementary material for: An option space approach to wood use: Providing structural timber for buildings while safeguarding forest integrity
Source: iScience. 2025 Sep 2;28(10):113472. doi: 10.1016/j.isci.2025.113472 (PMC12570369; doi:10.1016/j.isci.2025.113472)

iScience, Volume *28*

Supplemental information

An option space approach to wood use: Providing structural timber for buildings while safeguarding forest integrity

Simone Gingrich, Sarah Matej, Karl-Heinz Erb, Helmut Haberl, Julia Le Noë, Lisa Kaufmann, Andreas Magerl, Anke Schaffartzik, Dominik Wiedenhofer, and Stefan Pauliuk

Extended Data Fig.1: Regional wood demand in the building sector as quantified in the RECC model scenarios. The flows reported here at the carbon layer (Mt C/yr) correspond to flows $f_{II}+f_{III}$ in the system definition (Fig. S1), which is the industrial roundwood that is manufactured into structural timber for use in buildings.

Regional industrial roundwood demand in the building sector across scenarios

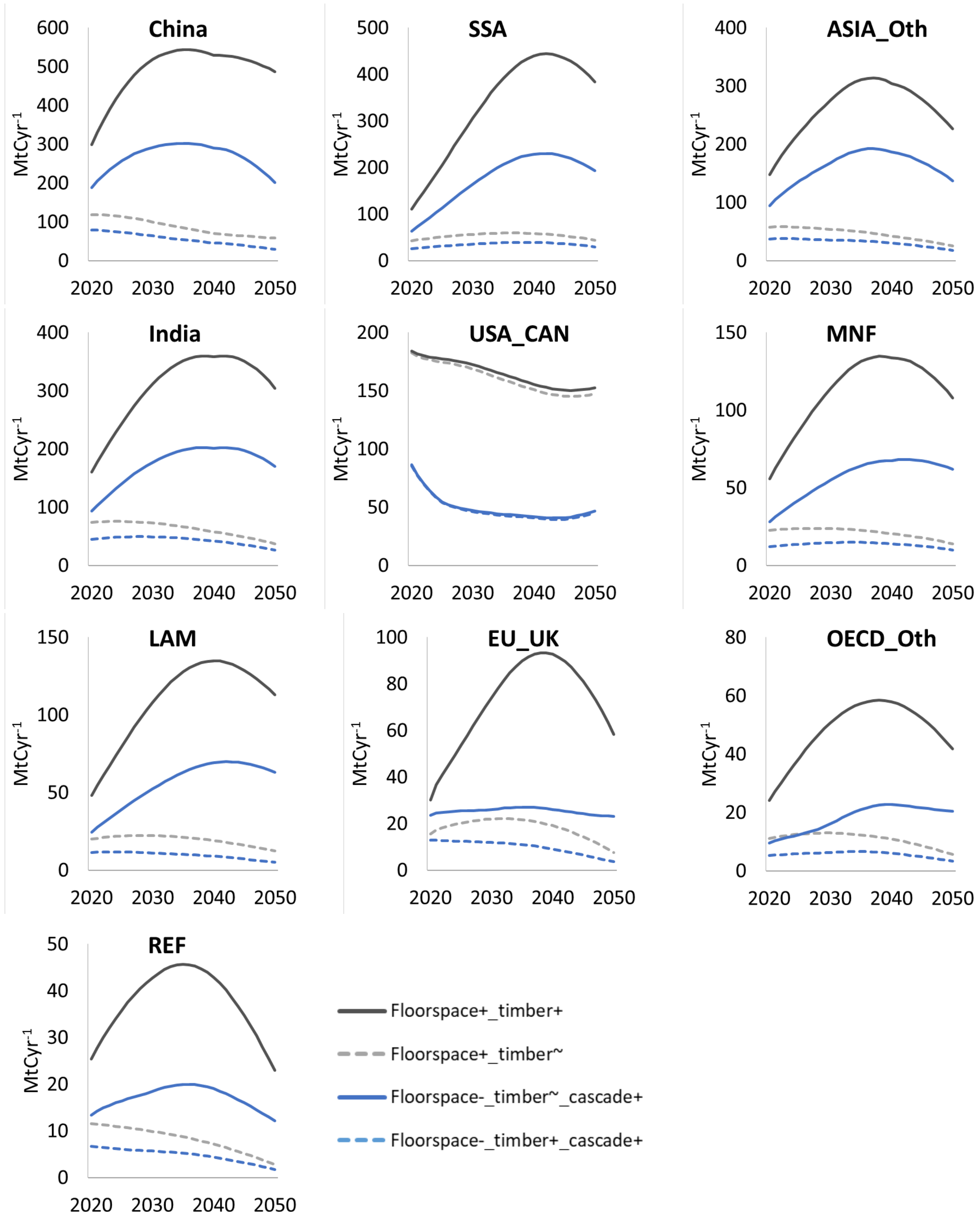

Extended Data Fig.2: Regional timber in-use stocks in buildings as quantified in the RECC model scenarios.

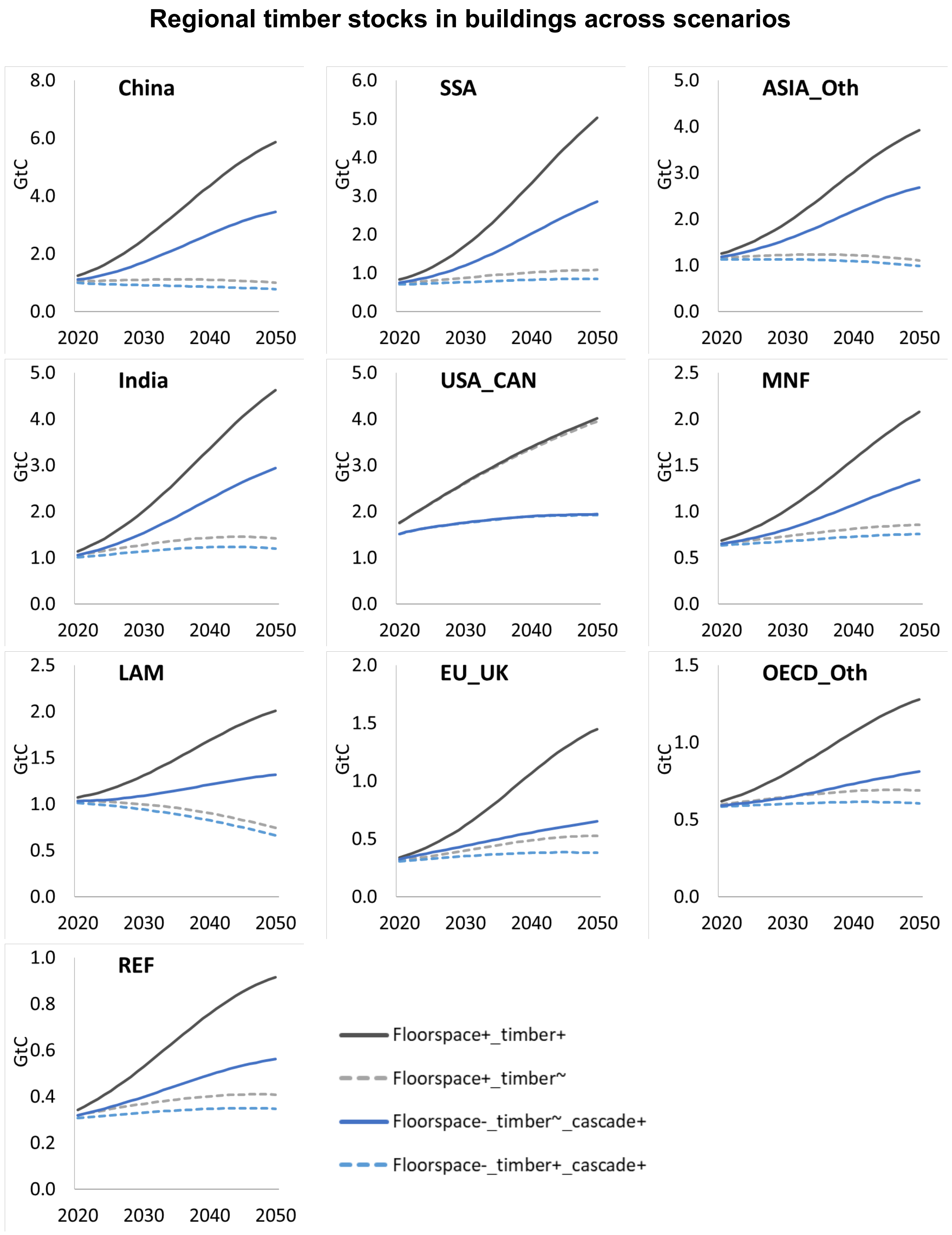

Extended Data Fig.3: Sensitivity of RECC scenario output to selected parameters, cumulative 2020-2050. a. industrial roundwood demand for use as structural timber in buildings, b. outflow of end-of-life structural timber from buildings ‘roundwood outflow’, c. timber in-use stock in buildings, d. system-wide GHG emissions.

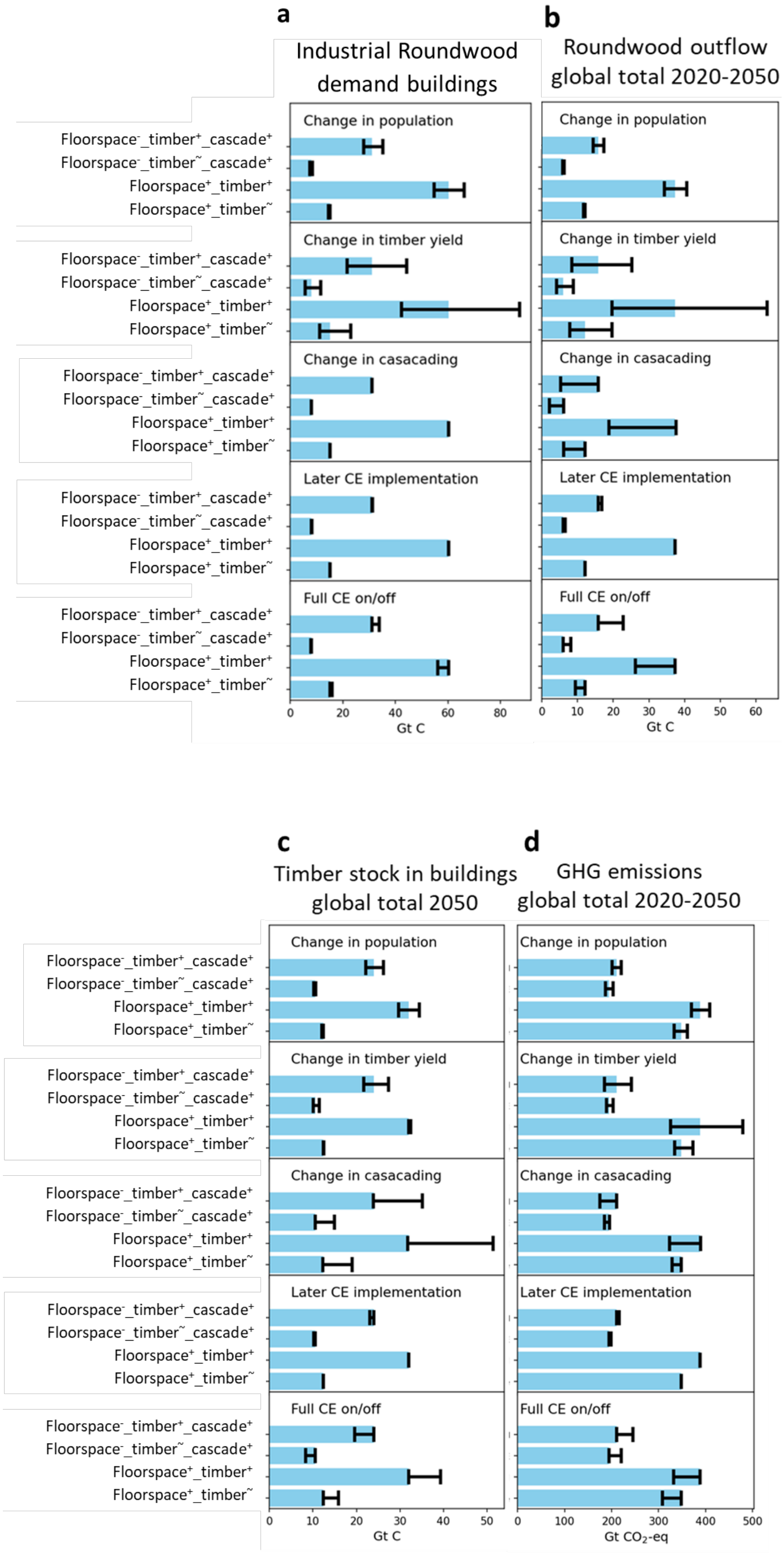

Extended Data Fig.4: Regional wood supply as quantified in the CRAFT model scenarios. The flow reported here at the carbon layer (Mt C/yr) corresponds to flow f_I in the system definition (Fig. S1).

Regional wood supply across scenarios

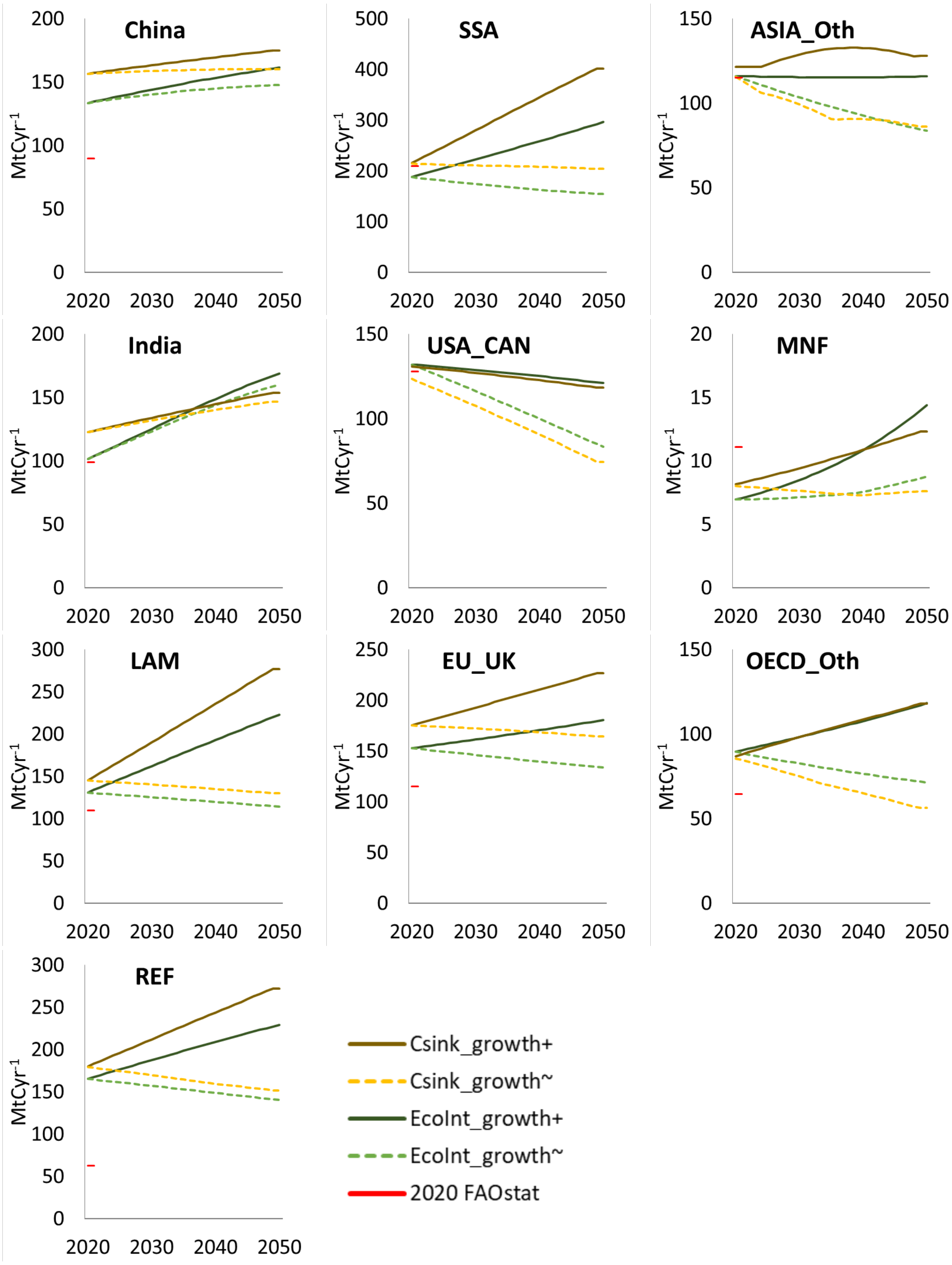

Extended Data Fig.5: Regional forest biomass stocks as quantified in the CRAFT model scenarios.

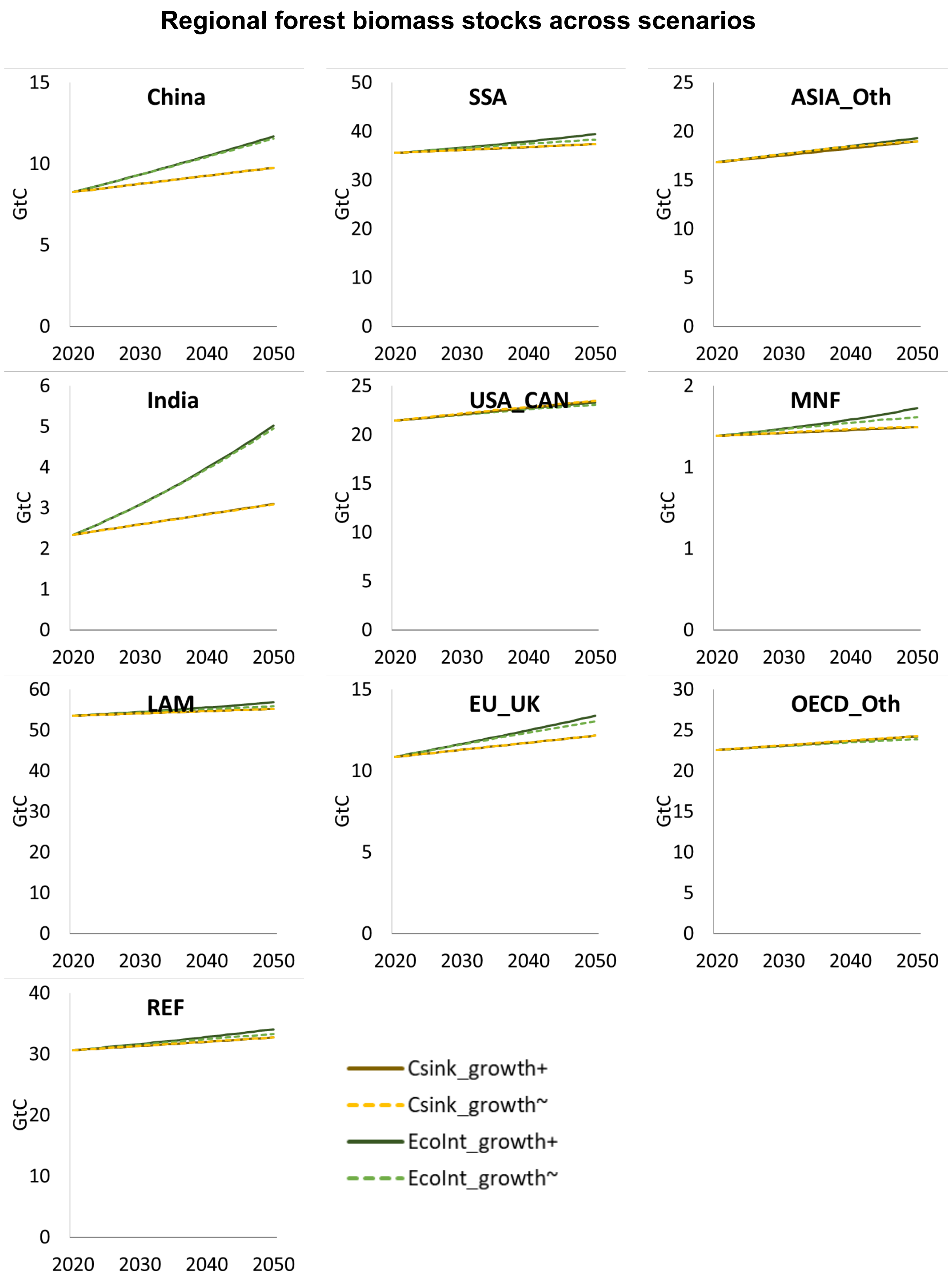

Extended Data Fig.6: Sensitivity of CRAFT model results on model input. r is the tree growth rate, α the change of r over time, see supplement for descriptions of the CRAFT model and the sensitivity analysis.

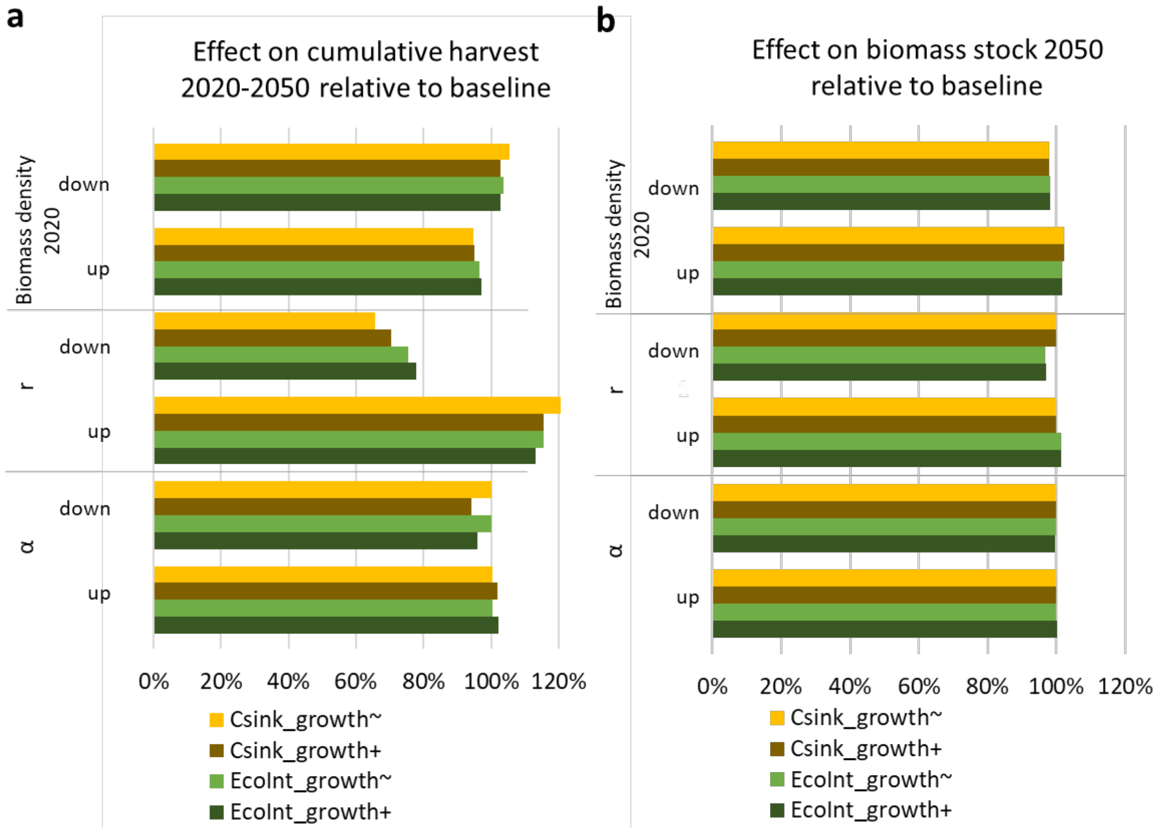

Supplement: Document S1. Extended Data Figures 1–6 [file mmc1.pdf]
