# Supplementary material for: An option space approach to wood use: Providing structural timber for buildings while safeguarding forest integrity
Source: iScience. 2025 Sep 2;28(10):113472. doi: 10.1016/j.isci.2025.113472 (PMC12570369; doi:10.1016/j.isci.2025.113472)
Supplement: Document S2. Figures S1–S3, Tables S1–S6, and supplement methods [file mmc2.pdf]

System definition for the CRAFT-RECC model coupling

Fig. S1 depicts the annual stocks and flows of C quantified in this analysis and specifies which variables are quantified in the RECC and CRAFT models, or via model coupling respectively. Annual timber harvest (f_I) is quantified by the CRAFT model, while RECC quantifies the industrial roundwood demand for structural timber (f_{II}+f_{III}). Those scenario combinations are considered feasible in which f_I quantified in the CRAFT model (i.e., the maximum ecologically allowable timber harvest) exceeds f_{II}+f_{III} quantified in the RECC model (i.e., the minimum threshold to provide sufficient structural timber quantities for the building material services).

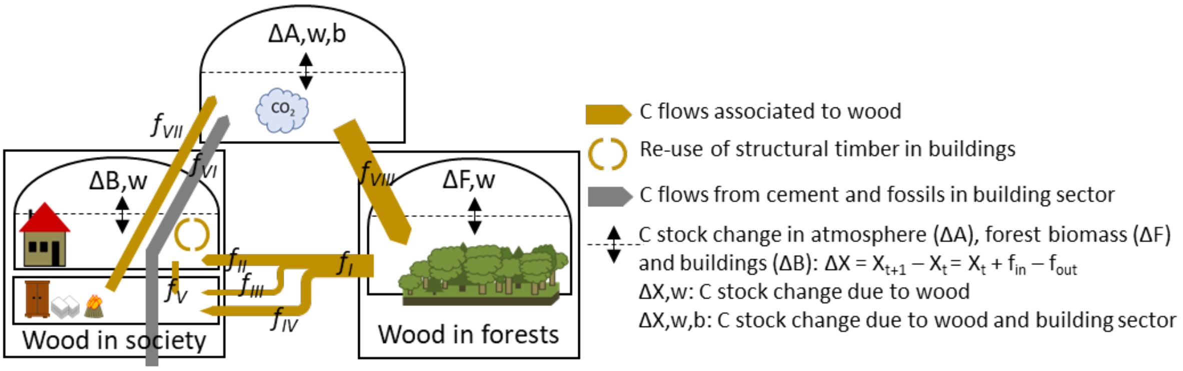

RECC model	CRAFT model	Model coupling
fI, fII, fIII, fV, fVI, fVII, ΔB,w	fI, fVIII, ΔF,w	fIV, ΔA,w,b

Fig. S1: *C stocks associated to wood use in society, ecosystems and atmosphere, and the annual C fluxes connecting them.*

The RECC model quantifies socio-economic demand for structural timber (f_{II}) based on assumptions of material service needs for shelter; losses during wood processing (f_{III}); the industrial roundwood harvest required to provide for this (f_{II}+f_{III}); timber stocks in buildings (ΔB,w); cascadic wood flows out of buildings into other applications (f_V); re-use of structural timber in buildings; and emissions from cement and fossil energy use in the building sector and upstream production (f_{VI}). Annual net biogenic carbon emissions from socio-economic wood use (f_{VII}) are given by the balancing equation of the building sector as f_{II}+f_{III} minus the difference between timber stocks in construction in two years.
The CRAFT model quantifies maximum allowable harvest (f_I) constrained by either annual forest biomass increase (ΔF,w; forest C sink target) or GAI (f_{VIII}; ecological integrity target). Primary wood available for socio-economic purposes other than construction (f_{IV}) is quantified as the difference between f_I and the sum of f_{II} and f_{III}. Atmospheric C pool change due to wood and building sector emissions (ΔA,w,b) is quantified as the sum of timber and cascaded wood products stock change in the construction sector, forest biomass stock change, and C flows from cement and fossils for construction: $\Delta A,w,c = \Delta C,w + \Delta F,w + f_{VI}$.

RECC model description

The resource efficiency–climate change (RECC)[1] mitigation model framework quantifies the material requirements for material service provision. RECC is based on dynamic material flow analysis and links the services provided (individual shelter and motorized transport) to the operation of in-use stocks of products (residential, non-residential buildings and passenger

vehicles), to their expansion and maintenance, and to their material cycles (Fig. S2) to model mitigation strategies and analyze trade-offs for environmental impacts along the products' life cycle. Here we use the shelter module of the model version 2.5[2].

RECC starts from material service scenarios, in our case per-capita m^2 in residential and non-residential buildings. To quantify material requirements for meeting these services, the model upscales product archetypes with different degrees of material and energy efficiency (13 residential and 24 non-residential building types), using engineering tools. This way, the model computes in-use stocks (e.g., timber in construction) and material and energy supply to provide these services (e.g., annual inflow of construction wood, annual energy demand of construction), as well as circular material flows (cascadic use of materials) and environmental impacts (greenhouse gas emissions). RECC operates at annual intervals and at the spatial resolution of ten world regions (Table S1). RECC scenarios are driven by parameters that augment the storylines of the shared socioeconomic pathways (SSP)[3] to describe future service demand and associated material requirements. In its current implementation (model version 2.5[2]), ten material efficiency strategies at different stages of the material cycle can be assessed individually or combined into bundles of strategies, by ramping up their implementation rates to the identified technical potentials. The model is implemented in Python and can be accessed at: https://www.industrialecology.uni-freiburg.de/odym-recc

Table S1*: Regions aggregated in this study. The country allocations are based on the SSP database https://tntcat.iiasa.ac.at/SspDb/dsd?Action=htmlpage&page=10#regiondefs. A full list of countries and their attribution to world regions is provided in the supplementary data.*

**Region name**	**Region description**
China	China
India	India
ASIA_Oth	Asian countries excluding China, India, and Japan
EU_UK	European Union member states and United Kingdom
OECD_Oth	OECD countries excluding European Union, USA and Canada
REF	Reforming economies of Eastern Europe, and the Former Soviet Union
MNF	Middle East and Northern Africa
SSA	Sub-Saharan Africa
USA_CAN	USA and Canada
LAM	Latin America

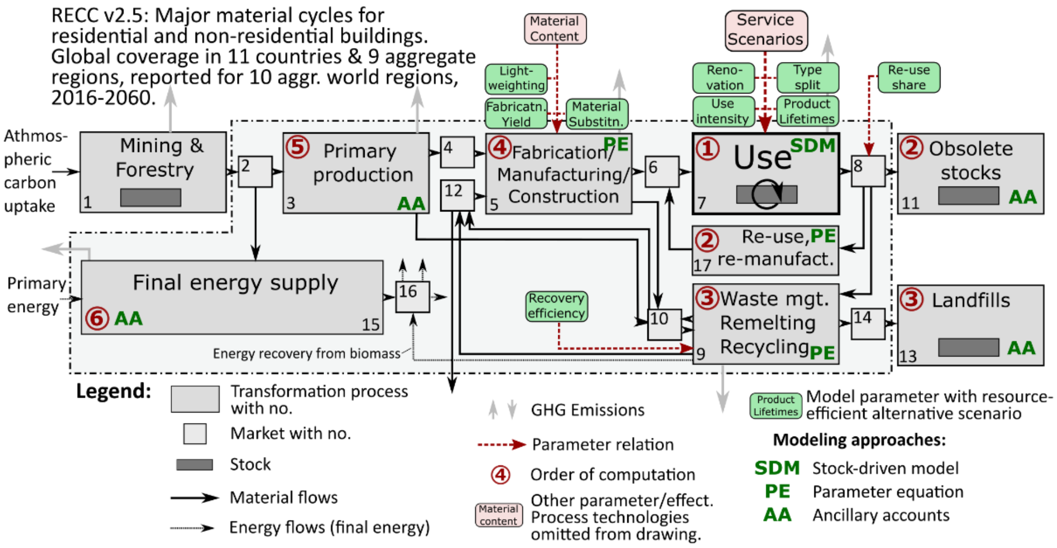

Fig. S2: RECC model system definition for the study of the global building stock, including construction wood. The flow of harvested wood, F_1_2, is the interface between the CRAFT and the RECC models.

For this analysis, wood cascade module flows and stocks were extracted from RECC's material flow module. The stocks, flows, and indicators related to biogenic carbon in the RECC model are summarized in the RECC carbon (C) cycle module system definition (Fig. S3) below.

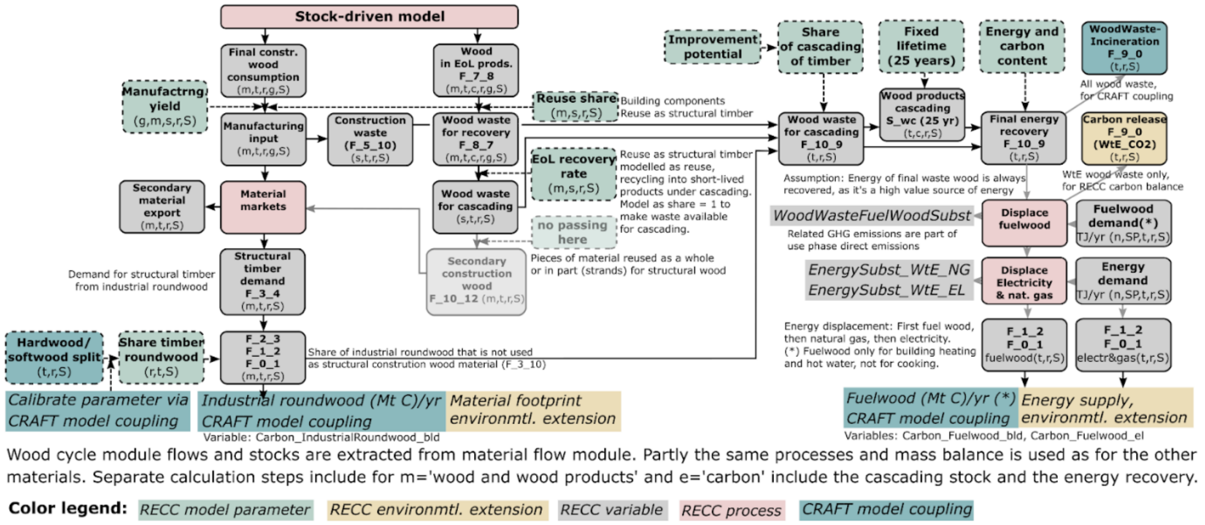

Fig. S3: The RECC carbon cycle module (update of Fig. 6.6 in Pauliuk 2023[2]).

The wood cascade/C cycle module is part of the RECC model equations[2] and included in the mass balance of the processes in the system definition for the chemical element C. The following four system variables are calculated for each scenario run and exported for the CRAFT-RECC model coupling:

- Wood from forests (carbon flow *F_1_2 ($f_{II}+f_{III}$ in the system definition in Fig. S1)*) enters the system as roundwood for being processed into timber in sawmills. This flow is a direct consequence of the demand for new construction and the timber intensity of new buildings ("construction wood demand").
- The total in-use stock S_7 of carbon in buildings and wood products in the use phase ("timber stocks in buildings").

- The total GHG impact of the system (Impacts_System_3579di (f_{VI} in the system definition in Fig. S1)) without carbon sequestration in forests. We subtract that part of *F_1_2* that enters the RECC system as fuelwood, because fuelwood emissions are already considered as forest biomass change in the CRAFT model output ("socio-economic GHG emissions from construction").
- The total wood outflow from the system after cascadic and energetic use, part of *F_9_0* ("roundwood outflow").

RECC scenarios on structural timber demand in buildings

The RECC model is driven by exogenous SSP-compatible population and service demand (in m²/capita) scenarios[4] scaled down to the model regions[5] (Table S1), scenarios on energy system future[6], assumptions on building type and energy carrier split that are derived from major scenario exercises[7], a number of efficient material production technologies, including those based on hydrogen, and a set of energy-material consistent building archetypes for different energy standards, light-weighting options, and wood use[8]. Details can be found in the RECC model parameter database (https://zenodo.org/uploads/12752350) and the model documentation[2].

Global demand for construction wood is driven by exogenous scenarios for per capita floor space by region and socio-economic scenario. The starting points for residential buildings are the scenario values developed for RECC v2.4.[5] Table S2 presents the per-capita floorspace assumptions applied in the two scenarios.

Table S2: *Central parameters for the stock-flow-service nexus of the use phase: Initial and future service level (per-capita floorspace) for the different socio-economic scenarios, and the typical building lifetime. Building lifetime can vary across age-cohorts and here, typical values are indicated.*

	**2015 per capita stock (m²)**		**2050 per capita stock, m², Floorspace⁻ / Floorspace⁺**		**Typical Building lifetime (yr)**	
**Regions**	**residential**	**non-res.**	**residential**	**non-res.**	**residential**	**non-res.**
SSA	11.4	0.8	19.4/33.4	7/12	50	45
LAM	34.4	3.0	30.3/44.3	7/12	50	45
EU_UK	37.7	12.5	31.2/46.2	12.8/20	100-180	60-80
China	36.1	10.8	31/50	13/20	27-40	30
India	11.7	0.8	25/38.1	7/12	50	45
Other_Asia	20.8	2.6	29.4/39	7.5/12.6	50	45
MNF	24.6	8.3	29.6/43.6	9/15	100	45
REF	23.5	5.9	29.5/43.5	9/15	120	60
Other_OEDC	38.0	6.5	30.5/44.5	9/15	100	50
USA_CAN	66.8	24.1	42.5/83.7	18/30	110	45

Energy supply and demand are modelled following the RCP2.6 low carbon future. Regarding materials, the extent of CE (narrow via light-weighting, slow, and close strategies) and wood use is specified individually for each scenario run (Table S3). If a certain material-related strategy is chosen, it is assumed to be ramped up to its currently identified technical potential

by 2035. Table S3 lists the 'corner scenarios' that span the demand side of the option space for wood supply and use for the CRAFT-RECC model coupling and results comparison.

Table S3: *Overview of the scenarios defined for the evaluation of the model results. All scenarios adhere to RCP 2.6.*

Scenarios	SSP	Description
Floorspace^{+}_timber$^{\sim}$	SSP2	SSP2 socio-economics, efficiency and low carbon renewable energy, no Circular Economy, low wood intensity in new buildings
Floorspace^{+}_timber^{+}	SSP2	SSP2 socioeconomics, efficiency and renewable energy, no Circular Economy, high wood intensity in new buildings
Floorspace^{-} _timber$^{\sim}$_cascade^{+}	LED	Low material and energy demand (LEMD), efficiency and renewable energy, Full Circular Economy, low wood intensity in new buildings
Floorspace^{-} _timber^{+}_cascade^{+}	LED	LEMD, efficiency and ren. energy, Full Circular Economy, high wood intensity in new buildings

Sensitivity analysis of RECC results

We tested the sensitivity of central RECC results with respect to changes in central input parameters that are known to affect timber demand and emissions from the timber cycle. Table S4 shows the parameter changes implemented and the rationale for choosing the alternative values. Relative to the four scenarios analysed (SSP2_Base, SSP2_Wood, LEMD_FullCE, LEMD_FullCE_Wood).

Table S4: *Overview of RECC parameter changes for the sensitivity analysis. For details, please see the log sheets of the different parameter files, archived under https://zenodo.org/uploads/12752350* .

Sensitivity case / Base scenario	Parameter(s) affected	Change in parameter	Rationale/Reference
Change in population	Population: 2_P_RECC_Population_SSP_32R_V2.3 changes to 2_P_RECC_Population_SSP_32R_V2.3 _SSP1_Sensitivity and 2_P_RECC_Population_SSP_32R_V2.3 _SSP3_Sensitivity	Low global population: SSP1 High global population: SSP3	Population is a key demand driver and varies over the SSP scenarios.[9]
Change in timber yield in sawmills	Timber from roundwood yield: 4_PY_TimberRoundWood_V1.1 to 4_PY_TimberRoundWood_V1.1_Low and 4_PY_TimberRoundWood_V1.1_High	High option: Set all values to highest value from literature: 0.7. Low option: Set all values to lowest value: 0.34.	Timber yield in sawmills is an uncertain parameter with direct consequences for the amount of timber produced, test for the full plausible range
Include FullCE for SSP2: SSP2_FullCE vs. SSP2_Base, SSP2_FullCE_Wood vs. SSP2_Wood, LEMD_FullCE vs. LEMD_Base, LEMD_FullCE_Wood vs. LEMD_Wood	All CE strategy parameters, see Pauliuk (2023).	All CE strategies rolled out to full extent by 2035.	Test for the impact of a full CE (esp. light-weighting and cascading) on timber demand.[2]

Change cascading of end-of-life wood<br>4 corner with low-base-high	4_PY_WoodCascading_V1.0 to 4_PY_WoodCascading_V1.0_Low and 4_PY_WoodCascading_V1.0_High<br>3_LT_Wood_Cascade_V1.0 to 3_LT_Wood_Cascade_V1.0_Long<br>(for the 'no cascading' option, lifetime is irrelevant)	No cascade: Share of wood going into cascade = 0.<br>More cascade: extent cascading lifetime from 25 to 40 years. Maximum cascading share from 0-30% to 50% for all regions.	Share of end-of-life wood going into another application (cascading) and lifetime of this use are uncertain parameters
Change speed of CE strategy implementation<br>4 corner with high alternative	3_SHA_RECC_REStrategyScaleUp_V3.4 to 3_SHA_RECC_REStrategyScaleUp_V3.4_Delayed	Delay the sinus-shaped ramp-up curve of CE strategy implementation so that 100% implementation is reach in 2050 instead of 2035.	Ramp-up time for CE strategies is inherently uncertain

For each sensitivity run, the database and config settings of the RECC v2.5 global building model are changed, e.g., by telling the model to use 4_PY_TimberRoundWood_V1.1_Low instead of 4_PY_TimberRoundWood_V1.1. With the modified database and model configuration, the entire list of scenario configurations (40 in total: four demand + CE scenarios for ten global regions) is re-run. The results for the carbon in the system variables *F_1_2, S_7,* and *F_9_0* are extracted from the result folders and compiled in a separate sheet by calling ODYM_RECC_Export_xlxs_Combine_Select.py, using the specifications given on sheet 'RECC_CRAFT_Sensitivity' in RECCv2.5_EXPORT_Combine_Select.xlsx. From the plotting specifications on the same sheet, the plotting script RECC_CRAFT_Sensitivity.py creates the figures that show the results of the sensitivity analysis. The model code is available via https://github.com/IndEcol/RECC-ODYM, the model commit ID used for the analysis presented here is dffb844.

CRAFT model description

The model CRAFT[10,11] was used to quantify wood harvest potentials under sustainability constraints for the period 2020 to 2050. Basis for this assessment was the reconstruction of global forest biomass stocks from 1990 to 2020, applying the approach described in Le Noë et al.[10], with the same input data for forest area and biomass stocks from FRA and a new download of wood harvest data from FAOSTAT (https://www.fao.org/faostat/en/#data/FO, "Production Quantity": "Wood-fuel, coniferous"; "Wood-fuel, non-coniferous"; "Industrial Roundwood, coniferous"; "Industrial Roundwood, non-coniferous") from April 2023. Following Le Noë et al.[10], we used coefficients from[12–14] to convert m^3 of wood to tC and account for residues, belowground biomass and losses.

The CRAFT model establishes a relationship between forest biomass stocks (B, $tCha^{-1}$) and annual forest growth (Net Primary Productivity, NPP, $tCha^{-1}yr^{-1}$) at the country level ($_c$) and annual resolution ($_t$):

1. $NPP_{c,t} = r_{c,t}B_{c,t}(1 - \frac{B_{c,t}}{K_c})$

With r denoting the forest growth rate parameter, and K the theoretical carrying capacity. These national-level values thus inform about the average forest characteristics in a country, considering all factors influencing forest growth including management and environmental conditions. The change in forest growth rate r over time is quantified in the linear change factor α:

2. $r_{c,t} = r_{c,1990} + \frac{(\alpha_c r_{c,1990} - r_{c,1990})}{(2020-1990)}(t - 1990)$

The parameters r, K and α are optimized at country level to accurately reconstruct data on forest biomass stock (B, $tCha^{-1}$) from 1990-2020, based on input data for forest area (A, ha), forest biomass harvest, including all woody and green biomass parts (H, $tCha^{-1}yr^{-1}$), as well as forest biomass mortality including vegetation fires and natural mortality (M, $tCha^{-1}yr^{-1}$):

3. $B_{c,t+1} = (B_{c,t} + NPP_{c,t} - H_{t+1} - M_{c,t}) \times MIN(1, \frac{A_t}{At+1})$

We used the Python function "scipy.optimize.differential_evolution" for the optimization, and applied the following constraints for the three parameters r (in 1990), K, and α: $0.03<r<0.21yr^{-1}$; $100<K<720\ tCha^{-1}$; and for α we modified a starting value derived from LPJ-GUESS (version without N limitation) simulations of changes in potential NPP in forests[15] for that period by ±0.06. We thus obtained model-based reconstructions for forest biomass change at national level for 1990-2020.

CRAFT scenarios on timber harvest supply

Using the CRAFT model, we quantified the maximum allowable harvest under specific ecological sustainability constraints. All scenarios assume a constant forest area (i.e., no deforestation or forest expansion), but vary in terms of (1) the ecological limit considered and (2) the expected conditions of future forest growth.

For (1), we define (a) an ecosystem integrity target in which management intensity[16] is limited such that gross wood extraction (i.e., harvest including residues, belowground biomass and losses) equals no more than 75% of annual increment in forests at the national level, and (b) a climate-change mitigation target derived from country-level C sequestration values from forest restoration from Roe et al.[17]. Values reported by Roe et al.[17] refer to the technical potential of

climate-change mitigation through forest management at the country level between 2020-2050[18,19]. As the target could not be reached in several countries, even with zero harvest, we defined aggregated regions, within which the target sinks could be traded. Within these regions we transferred the sink target that could not be reached in one country to other countries, starting with the country in which the most additional sink potential could be reached with the least reduction of wood harvest.

For (2), we applied "best" and "worst case" assumptions for future forest growth, denoted as "_imp", and "_noimp" scenarios. In the _imp scenario variants we assume that (a) improved management[20] or expansion of tree plantations in existing forests, and/or vegetation greening[21] result in continuously increasing forest growth rates in those countries where an increase in this parameter was observed in the model parametrization period of 1990-2020, and in stable growth rates in those countries where it declined in 1990-2020. This means that in countries where α_c was above 1 in the model parametrisation period 1990-2020, we applied the same α_c values for the period 2020-2050, and when it was below 1, we set it to 1 for 2020-2050 respectively. In the _noimp scenario variants, we assumed (b) vegetation browning[22] and/or increased disturbances due to future climate impacts[23] to lead to stable growth rates in countries where forest growth rates grew in 1990-2020 (i.e., if $\alpha_c>1$ in 1990-2020, we set it to 1 in 2020-2050), and to continuously declining growth rates in countries where it already declined in 1990-2020 (i.e., if $\alpha_c<1$ in 1990-2020, we use this value for 2020-2050). Fig. S3 displays the values for the CRAFT parameter r applied in the _imp and _noimp scenario variants respectively, at the country level for each world region, see also data supplement.

Fig. S3: *CRAFT model parameter r in different scenarios*

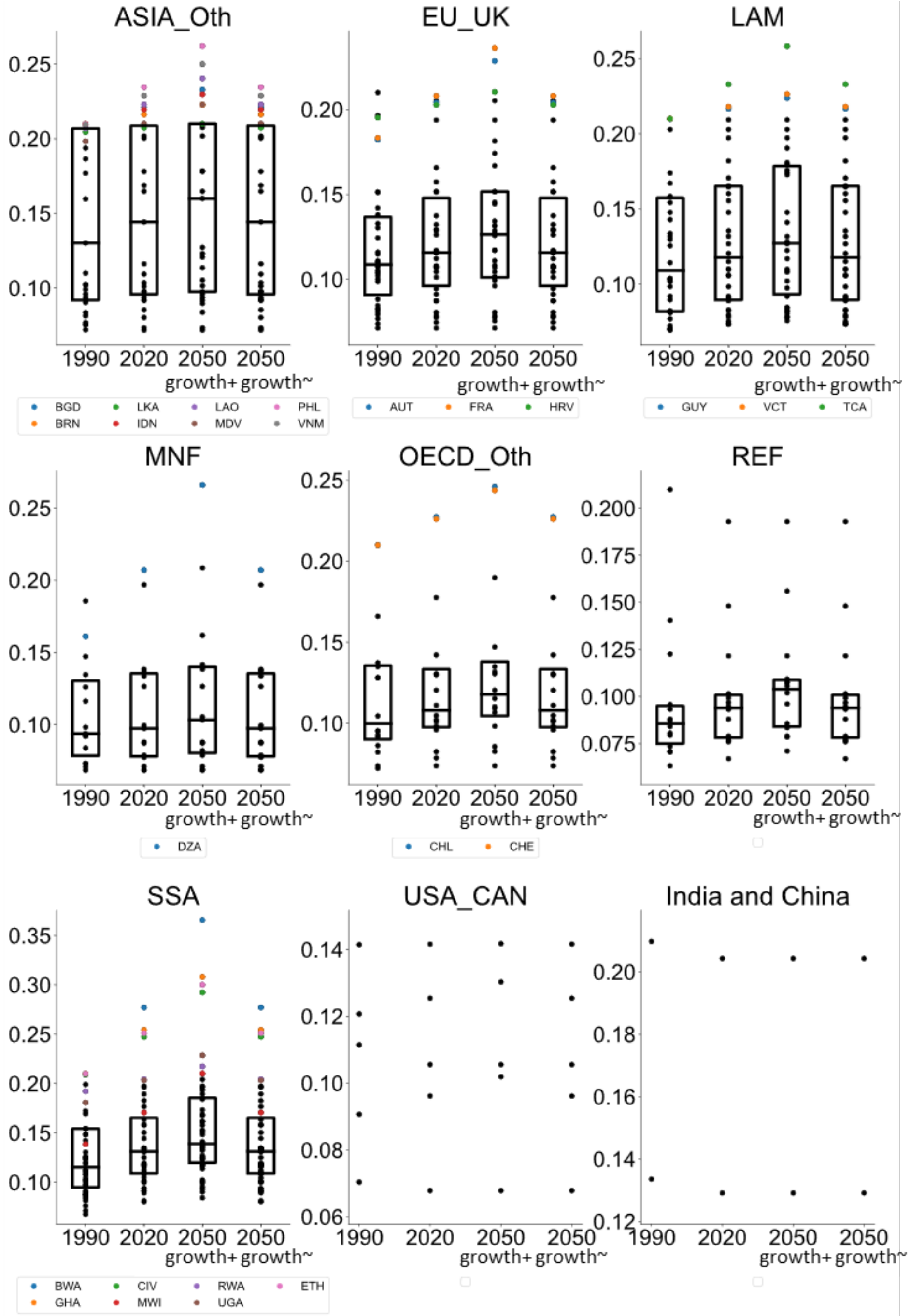

Based on the limits implemented, we quantified annual allowable harvest values (i.e., wood supply), as well as forest biomass C stocks for the period 2020-2050. National-level values of wood supply were aggregated to the eight world regions defined in the RECC model.

Sensitivity analysis of CRAFT results

We tested the sensitivity of the CRAFT results to changes in 3 parameters, namely r, α and the biomass density of 2020. Likely ranges of r and α were derived based on the reliability of countries' reports on C in biomass from FRA[24]. We used the proportion of forest area in data-reliability tier 3 for C assessments per world region (table 88 in[24]) and the proportion of global

area in tier 2 and tier 1 (table 87 in[24]) for 2020 and assumed the associated error in C estimates to be 2%, 4% and 6% for tier 3 to 1 respectively (see Table S5).

***Table S5**: Derived error associated with FRA estimates for C in forest biomass B for 2020*

	**% of forest area**			**average error in carbon stock estimation**
	**tier 3 (error: 2%)**	**tier 2: (error: 4%)**	**tier1: (error: 6%)**	
North and Central America	97%	2%	1%	2.1%
South America	66%	20%	14%	3.0%
Europe	90%	6%	4%	2.3%
Africa	1%	58%	41%	4.8%
Asia	18%	48%	34%	4.3%
global	61%	38%	1%	2.8%

By applying the resulting average uncertainty ranges of C accounts in FRA per world region to the CRAFT biomass density parametrization data, we optimized country-level r and α values for 1990 to 2020 for two additional runs: one "maximum increase", where the 1990 C density values were assumed to be over-estimated and therefore were corrected downwards and the 2020 values were corrected upwards; and another "maximum decline" with the reverse modification. The correction factor was linearly interpolated for the C density values reported between 1990 and 2020. We thus established two additional optimizations for r and α parameters at country level, which were once below and once above the parameters optimized for the baseline C density calibration data (Table S6).

***Table S6**: Ranges for sensitivity tests for biomass density and CRAFT parameters r and α derived based on FRA C density reliabilities, presented here as global averages weighted by forest biomass in 2020*

	**biomass density B [$tCha^{-1}$]**	**r [yr^{-1}]**	**α [%]**
**baseline**	87.5	0.117	1.062
**down**	84.6	0.113	1.048
**up**	90.4	0.122	1.078
**down [%baseline]**	97%	96%	99%
**up [%baseline]**	103%	104%	102%

The 6 alternative input parameters (Min and Max values for B, r, and α, respectively) were then used for each of the 4 wood scenarios modelled with CRAFT to quantify harvest and biomass trajectories from 2020 to 2050. The resulting variations in B, r, and α allow us to test to which extent our modelling results are sensitive to empirical uncertainties in reported biomass density, and observed forest growth, as well as to future potential changes in forest growth, which may e.g. stem from environmental change, change in management, or change in spatial distribution of forests, even if total forest area remains constant.

References

1. Pauliuk, S., Fishman, T., Heeren, N., Berrill, P., Tu, Q., Wolfram, P., and Hertwich, E.G. (2021). Linking service provision to material cycles: A new framework for studying the resource efficiency–climate change (RECC) nexus. J of Industrial Ecology *25*, 260–273. https://doi.org/10.1111/jiec.13023.

2. Pauliuk, S. (2023). Documentation of the RECC model v2.5 : open dynamic material systems model for the Resource Efficiency-Climate Change (RECC) Nexus (Albert-Ludwigs-Universität Freiburg).

3. Riahi, K., Van Vuuren, D.P., Kriegler, E., Edmonds, J., O'Neill, B.C., Fujimori, S., Bauer, N., Calvin, K., Dellink, R., Fricko, O., et al. (2017). The Shared Socioeconomic Pathways and their energy, land use, and greenhouse gas emissions implications: An overview. Global Environmental Change *42*, 153–168. https://doi.org/10.1016/j.gloenvcha.2016.05.009.

4. O'Neill, B.C., Kriegler, E., Ebi, K.L., Kemp-Benedict, E., Riahi, K., Rothman, D.S., Van Ruijven, B.J., Van Vuuren, D.P., Birkmann, J., Kok, K., et al. (2017). The roads ahead: Narratives for shared socioeconomic pathways describing world futures in the 21st century. Global Environmental Change *42*, 169–180. https://doi.org/10.1016/j.gloenvcha.2015.01.004.

5. Fishman, T., Heeren, N., Pauliuk, S., Berrill, P., Tu, Q., Wolfram, P., and Hertwich, E.G. (2021). A comprehensive set of global scenarios of housing, mobility, and material efficiency for material cycles and energy systems modeling. J of Industrial Ecology *25*, 305–320. https://doi.org/10.1111/jiec.13122.

6. PBL (2024). Welcome to IMAGE 3.2 Documentation - IMAGE.

7. IEA (2022). World Energy Outlook 2022.

8. Krych, K., and Heeren, N. (2024). BuildME: A combined energy–material simulation framework for modeling resource efficiency in buildings. Journal of Industrial Ecology.

9. Kc, S., and Lutz, W. (2017). The human core of the shared socioeconomic pathways: Population scenarios by age, sex and level of education for all countries to 2100. Global Environmental Change *42*, 181–192. https://doi.org/10.1016/j.gloenvcha.2014.06.004.

10. Le Noë, J., Erb, K.-H., Matej, S., Magerl, A., Bhan, M., and Gingrich, S. (2021). Altered growth conditions more than reforestation counteracted forest biomass carbon emissions 1990–2020. Nat Commun *12*, 6075. https://doi.org/10.1038/s41467-021-26398-2.

11. Le Noë, J., Matej, S., Magerl, A., Bhan, M., Erb, K., and Gingrich, S. (2020). Modeling and empirical validation of long-term carbon sequestration in forests (France, 1850–2015). Glob Change Biol *26*, 2421–2434. https://doi.org/10.1111/gcb.15004.

12. Haberl, H., Erb, K.H., Krausmann, F., Gaube, V., Bondeau, A., Plutzar, C., Gingrich, S., Lucht, W., and Fischer-Kowalski, M. (2007). Quantifying and mapping the human appropriation of net primary production in earth's terrestrial ecosystems. Proceedings of the National Academy of Sciences *104*, 12942–12947. https://doi.org/10.1073/pnas.0704243104.

13. Kastner, T., Matej, S., Forrest, M., Gingrich, S., Haberl, H., Hickler, T., Krausmann, F., Lasslop, G., Niedertscheider, M., Plutzar, C., et al. (2022). Land use intensification increasingly drives the spatiotemporal patterns of the global human appropriation of net primary production in the last century. Global Change Biology *28*, 307–322. https://doi.org/10.1111/gcb.15932.

14. Krausmann, F., Erb, K.-H., Gingrich, S., Haberl, H., Bondeau, A., Gaube, V., Lauk, C., Plutzar, C., and Searchinger, T.D. (2013). Global human appropriation of net primary production doubled in the 20th century. Proceedings of the National Academy of Sciences *110*, 10324–10329. https://doi.org/10.1073/pnas.1211349110.

15. Kastner, T., Matej, S., Forrest, M., Gingrich, S., Haberl, H., Hickler, T., Krausmann, F., Lasslop, G., Niedertscheider, M., Plutzar, C., et al. (2022). Land use intensification increasingly drives the spatiotemporal patterns of the global human appropriation of net primary production in the last century. Global Change Biology *28*, 307–322. https://doi.org/10.1111/gcb.15932.

16. Oettel, J., and Lapin, K. (2021). Linking forest management and biodiversity indicators to strengthen sustainable forest management in Europe. Ecological Indicators *122*, 107275. https://doi.org/10.1016/j.ecolind.2020.107275.

17. Roe, S., Streck, C., Beach, R., Busch, J., Chapman, M., Daioglou, V., Deppermann, A., Doelman, J., Emmet-Booth, J., Engelmann, J., et al. (2021). Land-based measures to mitigate climate change: Potential and feasibility by country. Global Change Biology *27*, 6025–6058. https://doi.org/10.1111/gcb.15873.

18. Austin, K.G., Baker, J.S., Sohngen, B.L., Wade, C.M., Daigneault, A., Ohrel, S.B., Ragnauth, S., and Bean, A. (2020). The economic costs of planting, preserving, and managing the world's forests to mitigate climate change. Nat Commun *11*, 5946. https://doi.org/10.1038/s41467-020-19578-z.

19. Griscom, B.W., Busch, J., Cook-Patton, S.C., Ellis, P.W., Funk, J., Leavitt, S.M., Lomax, G., Turner, W.R., Chapman, M., Engelmann, J., et al. (2020). National mitigation potential from natural climate solutions in the tropics. Phil. Trans. R. Soc. B *375*, 20190126. https://doi.org/10.1098/rstb.2019.0126.

20. Kaarakka, L., Cornett, M., Domke, G., Ontl, T., and Dee, L.E. (2021). Improved forest management as a natural climate solution: A review. Ecol Sol and Evidence *2*, e12090. https://doi.org/10.1002/2688-8319.12090.

21. Zhang, Y., Song, C., Band, L.E., Sun, G., and Li, J. (2017). Reanalysis of global terrestrial vegetation trends from MODIS products: Browning or greening? Remote Sensing of Environment *191*, 145–155. https://doi.org/10.1016/j.rse.2016.12.018.

22. Pan, N., Feng, X., Fu, B., Wang, S., Ji, F., and Pan, S. (2018). Increasing global vegetation browning hidden in overall vegetation greening: Insights from time-varying trends. Remote Sensing of Environment *214*, 59–72. https://doi.org/10.1016/j.rse.2018.05.018.

23. Seidl, R., Thom, D., Kautz, M., Martin-Benito, D., Peltoniemi, M., Vacchiano, G., Wild, J., Ascoli, D., Petr, M., Honkaniemi, J., et al. (2017). Forest disturbances under climate change. Nature Clim Change *7*, 395–402. https://doi.org/10.1038/nclimate3303.

24. FAO (2020). Global Forest Resources Assessment 2020: Main report (FAO) https://doi.org/10.4060/ca9825en.
